# The Effects of Theta Precession on Spatial Learning and Simplicial Complex Dynamics in a Topological Model of the Hippocampal Spatial Map

**DOI:** 10.1371/journal.pcbi.1003651

**Published:** 2014-06-19

**Authors:** Mamiko Arai, Vicky Brandt, Yuri Dabaghian

**Affiliations:** 1 The Jan and Dan Duncan Neurological Research Institute, Baylor College of Medicine, Houston, Texas, United States of America; 2 Department of Computational and Applied Mathematics, Rice University, Houston, Texas, United States of America; University of Pittsburgh, United States of America

## Abstract

Learning arises through the activity of large ensembles of cells, yet most of the data neuroscientists accumulate is at the level of individual neurons; we need models that can bridge this gap. We have taken spatial learning as our starting point, computationally modeling the activity of place cells using methods derived from algebraic topology, especially persistent homology. We previously showed that ensembles of hundreds of place cells could accurately encode topological information about different environments (“learn” the space) within certain values of place cell firing rate, place field size, and cell population; we called this parameter space the learning region. Here we advance the model both technically and conceptually. To make the model more physiological, we explored the effects of theta precession on spatial learning in our virtual ensembles. Theta precession, which is believed to influence learning and memory, did in fact enhance learning in our model, increasing both speed and the size of the learning region. Interestingly, theta precession also increased the number of spurious loops during simplicial complex formation. We next explored how downstream readout neurons might define co-firing by grouping together cells within different windows of time and thereby capturing different degrees of temporal overlap between spike trains. Our model's optimum coactivity window correlates well with experimental data, ranging from ∼150–200 msec. We further studied the relationship between learning time, window width, and theta precession. Our results validate our topological model for spatial learning and open new avenues for connecting data at the level of individual neurons to behavioral outcomes at the neuronal ensemble level. Finally, we analyzed the dynamics of simplicial complex formation and loop transience to propose that the simplicial complex provides a useful working description of the spatial learning process.

## Author Summary

One of the challenges in contemporary neuroscience is that we have few ways to connect data about the features of individual neurons with effects (such as learning) that emerge only at the scale of large cell ensembles. We are tackling this problem using spatial learning as a starting point. In previous work we created a computational model of spatial learning using concepts from the field of algebraic topology, proposing that the hippocampal map encodes topological features of an environment (connectivity) rather than precise metrics (distances and angles between locations)—more akin to a subway map than a street map. Our model simulates the activity of place cells as a rat navigates the experimental space so that we can estimate the effect produced by specific electrophysiological components —cell firing rate, population size, etc.—on the net outcome. In this work, we show that *θ* phase precession significantly enhanced spatial learning, and that the way downstream neurons group cells together into coactivity windows exerts interesting effects on learning time. These findings strongly support the notion that theta phase precession enhances spatial learning. Finally, we propose that ideas from topological theory provide a conceptually elegant description of the actual learning process.

## Introduction

Considerable effort has been devoted over the years to understanding how the hippocampus is able to form an internal representation of the environment that enables an animal to efficiently navigate and remember the space [1]. This internal map is made possible, in part, by the activity of pyramidal neurons in the hippocampus known as place cells [2], [3]. As an animal explores a given environment, different place cells will fire in different, discrete regions of the space that are then referred to as that cell's “place field” [2], [3]. Despite decades of research, however, the features of the environment that are encoded, the identity of the downstream neurons that decode the information, and how the spiking activity of hundreds of cells is actually used to form the map all remain unclear.

We recently developed a computational model for spatial learning, focusing on what information is available to the still-unidentified downstream neurons [4]. We reasoned that the information they decode must be encapsulated in the temporal patterns of the place cell spike trains, specifically place cell co-firing [4], [5]. Because place cell co-firing implies that the respective place fields overlap, the resulting map should derive from a sequence of overlaps between parts of the environment. The information encoded by the hippocampus would therefore emphasize connectivity between places in the environment, which is a topological rather than a geometric quality of space [4]. One advantage of this line of reasoning is that a topological problem should be amenable to topological analysis, so we developed our model using conceptual tools from the field of algebraic topology and, in particular, persistent homology theory [6], [7]. We simulated a rat exploring several topologically distinct environments and found that the information encoded by place cell co-firing can, in fact, reproduce the topological features of a given spatial environment. We also found that, in order to form an accurate spatial map within a biologically reasonable length of time, our simplified model hippocampus had to function within a certain range of values that turned out to closely parallel those obtained from actual experiments with healthy rodents. We called this sweet spot for spatial learning the learning region, L [4]. As long as the values of the three parameters (firing rates, place field sizes, and number of active neurons) remain within the learning region, spatial learning is reliable and reproducible. Beyond the perimeters of L, however, spatial learning fails.

Several features of this model are intuitively appealing. First, the size and shape of L vary with the difficulty of the task: the greater the complexity of the space to be learned, the narrower the range of values that can sustain learning and thus the more compact the learning region. Second, there is a certain tolerance for variation among the three parameters within L: if one parameter begins to fall outside the sweet spot, spatial learning can still occur if there is sufficient compensation in the other two parameters. Our model suggests that certain diseases (e.g., Alzheimer's) or environmental toxins (e.g., ethanol, cannabinoids) disrupt spatial learning over time by gradually shifting mean neuronal function (place cell firing, neuronal number, or place field size) beyond the perimeter of the learning region. This notion receives support from studies of mouse models that show a correlation between impairment in spatial cognition and larger, more diffuse place fields, lower place cell firing rates, and smaller numbers of active cells [8], [9]. All this corresponds well with our subjective experiences of learning: the complexity of the task influences learning time; when the task is difficult we can feel we are at or just beyond the limits of our capacity; disease or intoxication can reveal limits in our spatial cognition that would normally be compensated for.

In this paper we focus on analyzing the structure of the learning region itself. We begin by making the computational model more physiologically accurate. There is a *θ* (theta) component of subcortical LFP oscillations that occurs in the frequency range of 6-12 Hz and regulates spiking activity [10]. The timing of place cell spiking in the hippocampus is coupled with the phase of *θ-*oscillations so that, as a rat progresses through a particular place field, the corresponding place cell discharges at a progressively earlier phase of each new *θ*-cycle [11]. This phenomenon, called theta phase precession, reproduces short sub-sequences of an animal's current trajectory during each *θ-*cycle [11]. This has been construed to suggest that *θ-*phase precession helps the hippocampus remember the temporal sequence of the rat's positions in a space (i.e., its trajectory) [12], [13], thereby enhancing spatial learning and memory. If this is the case, *θ* phase precession should enhance learning in our computational model. Indeed, we find that it significantly improves and stabilizes spatial learning. We also find that different temporal windows to define co-firing exert a pronounced influence on learning time, and the most efficacious window widths correspond with experimental predictions. Finally, we analyze simplicial complex formation within the learning region, examining both the structure of the complexes and the dynamics of loop formation, and find an explanation for the poor efficiency of ensembles at the boundary of the learning region compared to peak-performing ensembles.

## Results

### Concepts underlying the topological model for spatial learning

**Figure 1:**
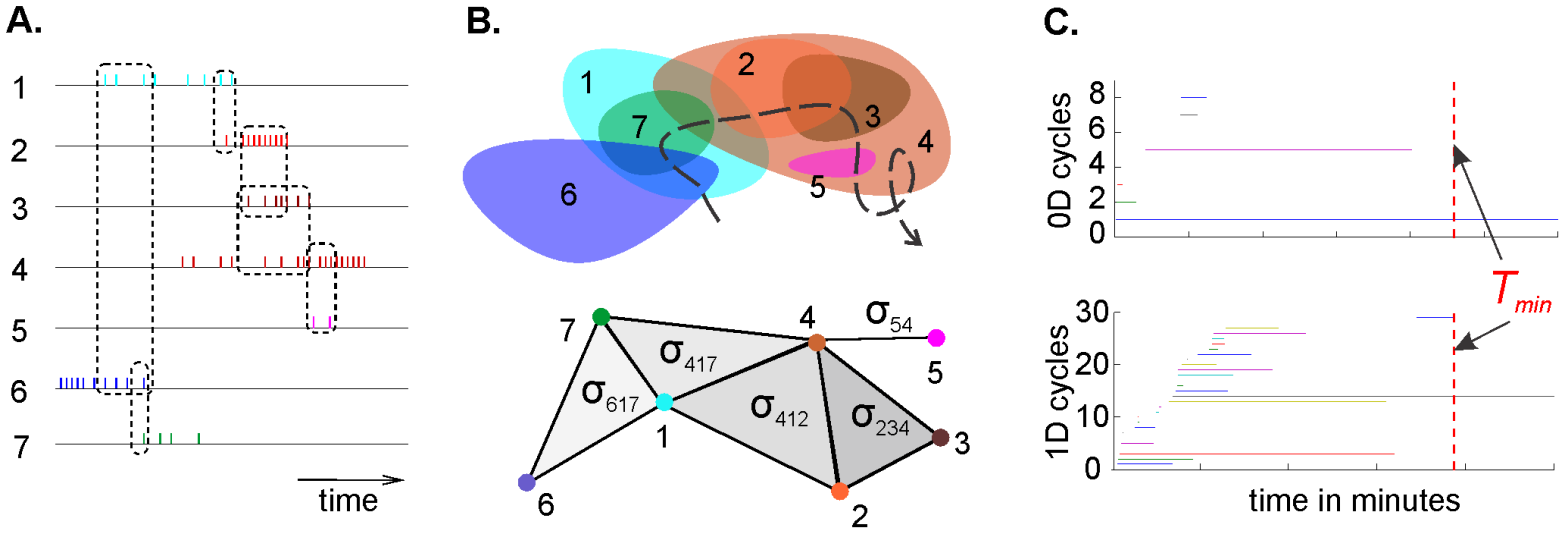
A simplicial complex can be derived from place cell co-firing. As an animal (in experiments, typically a rodent) explores a space, place cells fire in discrete locations that are mapped onto the space as place fields (**b**, colored ovals). (**A**) Shown are seven place cells firing, with some temporal overlap. (**B**) *Top*: The seven corresponding place fields, along with a fragment of an animal's trajectory (dashed line). *Bottom:* The elements of the nerve (a.k.a. Čech) simplicial complex generated by the overlaps among place fields. To form a simplicial complex, each place field center is considered to be a vertex, and each link between vertices is a simplex. Each simplex *σ_ij_* or *σ_ijk_* is labeled to indicate the vertices linked, e.g., σ_617_ indicates a link between vertices 6,1 and 7. (**C**) Persistent homology “barcodes” show the timelines of 0*D* and 1*D* loops, respectively: each colored horizontal line represents one 0*D* loop (top panel) or one 1*D* loop (bottom panel). The time *T_min_* (dotted red vertical lines) marks the moment when spurious loops (topological ‘noise’) disappear and the correct number of loops persists, in this case one in 0*D* and one in 1*D*, indicating that there is one hole in the environment. Thus, *T_min_* is the time after which the correct topological information emerges, which corresponds to the map formation or learning time in this environment, for this particular ensemble of place cells, operating under particular conditions of mean firing rate, mean place field size, number of cells in the population.

We will first briefly describe the fundamental concepts on which our model is based (this section is an abbreviated version of the approach described in [4]). Central to this work is the concept of a nerve simplicial complex, in which a space *X* is covered by a number of smaller, discrete regions [14]. If two regions overlap, the corresponding vertices, *v_i_* and *v_j_*, are considered connected by a 1*D* link *v_ij_* (**Figure 1**). If three regions overlap, then *v_ij_*, *v_jk_*, and *v_ki_* support a 2*D* triangular facet or simplex *σ_ijk_*, and so on as the number of overlaps and links increase. The structure of the simplicial complex approximates the structure of the environment: the complex N(*X*) obtained from a sufficiently dense cover of the space *X* will reproduce the correct topological indices of *X* (see [4] for details). For our model we developed a temporal analogue to the simplicial complex, i.e., a simplicial complex that builds over time: when the animal is first introduced to the environment, there will be only a few data points from place cell firing, but as the animal explores the space the place cell firing data accumulate. (Rodent experiments indicate that place fields take about four minutes to develop [15].) As the animal explores its environment and more place cells fire (and co-fire), the simplicial complex T grows with *T* (time) (T = T(*T)*). Eventually, after a certain minimal time *T*_min_, the space's topological characteristics will stabilize and produce the correct topological indices, at which point the topological information is complete. *T_min_* is thus the minimal learning time, the time at which a topologically correct map is first formed.

The correct topological indices are indicated by Betti numbers, which in turn are manifested in persistent cycles (see [4], [7], [16]). As the rat begins to explore an environment, the simplicial complex T(*T*) will consist mostly of 0-cycles that correspond to small groups of cofiring cells that mark contractible spatial domains. As the rat continues to explore the environment, the co-firing cells will produce links between the vertices of T(*T*), and higher-dimensional cycles will appear. As *T* increases, most cycles in each dimension will disappear as so much “topological noise,” leaving only a few persisting cycles that express stable topological information (**Figure 1C**). The persistent homology method [6] (see [4] **Methods**) enables us to distinguish between cycles that persist across time (reflecting real topological characteristics) and transient cycles produced by the rat's behavior (e.g., circling in a particular spot during one trial or simply not venturing into one part of the space during early explorations). The pattern of cycles is referred to as a barcode [16] that can be easily read to give topological information about a given environment (**Figure 1C**) [6], [7].

### Theta precession enhances spatial learning

If theta precession serves to enhance learning, as has been predicted [17]–[19], then it should enhance spatial learning in our model. This could occur by any of several means. First, theta precession might enlarge the number of ensembles capable of the task by expanding the scope of the parameters (including firing rates or place field sizes normally out of the bounds of L). Second, it might make the ensembles that are in L converge on the correct topological information more rapidly. Third, it might make the same ensembles perform more reliably (e.g., succeeding in map formation a greater percentage of times in our simulations).

To test the effect of theta precession in our model, we compared the rates of map formation for those formed with and without *θ-*precession. We tested 1710 different place cell ensembles by independently varying the number of place cells (*N*; 19 independent values, from 50 to 500), the ensemble mean firing rate (*f*; 10 independent values, from 4 to 40), and the ensemble mean place field sizes (*s*; 9 independent values, from 5 to 30) [Methods; see [4] and Methods therein for further details]. For statistical analysis, we simulated each map 10 times so that we could compute the mean learning time and its relative variability, *ξ* = Δ *T_min_*/*T_min_*, for each set of (*s*, *f*, *N*) values. In the following we will suppress the bar in the notation for the mean *f, s, N,* and *T_min_*.

**Figure 2:**
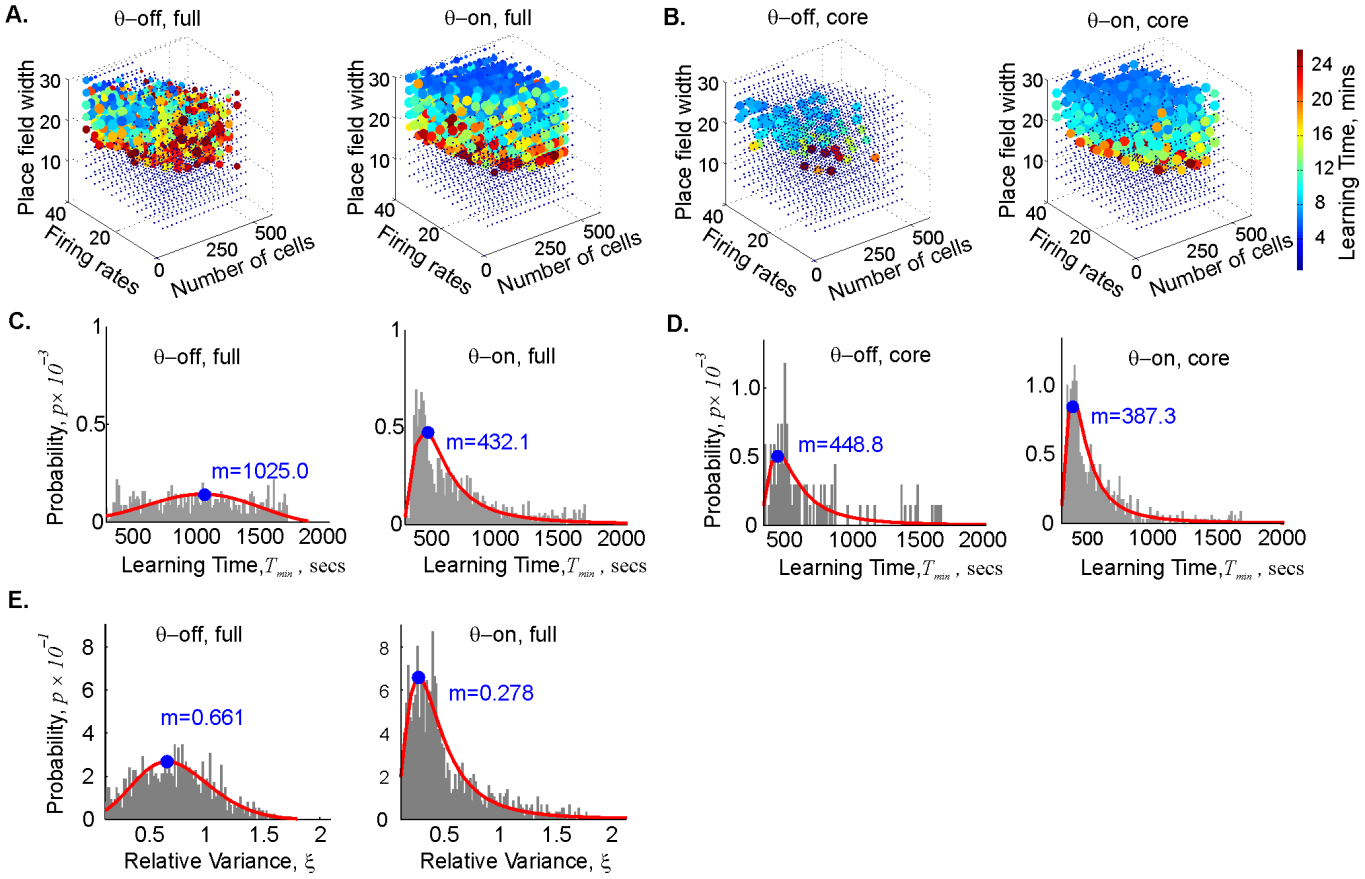
Theta precession both enlarges the learning region and reduces mean learning times. (**A**) Point clouds representing the mean learning times *T_min_* computed for the *θ*-off case (left) and the maps driven by a *θ*–signal recorded in rat (right). Each point corresponds to a place cell ensemble with a specific number of place cells, *N*, the mean ensemble firing rate, *f*, the mean ensemble place field size *s*. Dark blue circles represent those ensembles that form correct topological maps most rapidly and reliably; as the color shades from blue through green, yellow, and red, the learning times increase and map formation becomes less reliable (see [4], Methods). The rat *θ*–signal enlarges the learning region L and speeds map formation. (**B**) To zero in on the effect of *θ* precession on the quality of learning, these clouds depict only the maps that converged at least 7 out of 10 times (*ρ* ≥0.7), and for which the variance of the learning times, *ξ* =  Δ*T_min_*/*T_min_*, did not exceed 30% of the mean value. Even in this more rigorously defined core of L, with ensembles that already function well, the *θ*–signal has a pronounced effect. (**C**) Histograms of the minimal times obtained in the *θ*-off (left) and the *θ*-driven case (right), fit by the GEV distribution. The blue dot marks the mode of the distribution; m in the center of each panel gives the value of the mode. All convergent maps are included. The typical learning time *T_min_* in the *θ*-on case is about half as long as in the *θ*-off case. (**D**) The same histograms obtained for the core of L (*ρ* ≥0.7, *ξ*≤0.3). The typical learning time *T_min_* in the *θ*-on case is about 6.5 minutes, whereas without *θ* it is 15% longer. (**E**) One of the major effects of *θ* phase precession is to reduce the variability of the learning times. The histograms show that the typical value of the relative variation *ξ* in the *θ*-on case is less than half that of the *θ*-off case, i.e., that repeated simulations of the *θ*-driven maps more reliably reproduce similar learning time values.

**Figure 2** shows the results of these simulations in a 1×1 m space with one hole. (The size of the environment in this study is smaller than the ones used in [4], for two reasons: to avoid the potential problem of place cells with more than one field, and to reduce computational cost; see Methods.) The learning region L is small and sparse in the *θ*-off case, but notably larger and denser in the *θ*-on case (**Figure 2A**). Values that would be just beyond the learning region—*N* that may be too small, or place fields that are too large or too small, or firing rates too high or too low [4]—thus become functional with the addition of *θ*-precession.

Two criteria reveal the quality of the map-forming ensembles: speed and consistency in converging toward the correct topological signature. The fastest map formation times (under 4 minutes) are represented by blue dots; as the color shifts toward red, map formation times become longer and the error rate (failure to converge) increases. The size of the dot represents the success rate: small dots represent ensembles that only occasionally converge on the correct information, large dots represent ensembles that converge most or all of the time. *θ-*precession increases the probability of convergence across all ensembles that can form accurate maps at all (**Supplemental Figure S1**). Since we were interested in understanding the dynamics of efficient learning, however, we created a more stringent definition of the learning region to focus on the core of L where map-formation is most rapid and reliable, as well as to make the results more legible (L can be quite dense, as in **Figure 2A** **and Supplemental Figure S1**); if *θ-*precession truly enhances learning, its effect should be apparent even in the most successful ensembles, and indeed this was the case. The point clouds in **Figure 2B** depict those ensembles that formed maps with a convergence rate of *ρ*≥0.7 (i.e., those that produced correct topological information at least 70% of the time) and simultaneously had low relative variability of the *T_min_* values, *ξ*<0.3. Even within this more efficient core of L, the effect of *θ-*precession was pronounced.

The histograms of the computed mean learning times are closely fit by the Generalized Extreme Value (GEV) probability distribution (**Figure 2B**). The distributions show that *θ-*precession reduced the mean learning times *T_min_*: the mode of all the *θ*-on GEV distributions decreased by ∼50% compared with the *θ*-off case for the learning region as a whole (**Figure 2C**) and by ∼15% for the efficient ensembles at the core of L (**Figure 2D**). Moreover, the effects of adding *θ-*precession—reducing map formation time and decreasing the relative variability of the *T_min_* values—were manifested in all maps, not just those with high (*ρ* ≥0.7) convergence rates (**Figure 2C,D**). The histograms for all maps (all *ρ* -values) fit by the GEV distribution reveal that the typical variability (the mode of the distributions) in the *θ*-on cases is about half the size of the *θ*-off case (**Figure 2E**). In our model, therefore, *θ*-precession strongly enhances spatial learning.

### Theta precession enhances learning regardless of specific theta rhythm

Since we do not know what features of *θ-*oscillations might be important [20], we studied four different *θ*-oscillations, two simulated and two derived from electrophysiological experiments in wild-type rodents. Specifically, we modeled the effect of theta precession on the topological map by coupling the place cells' Poisson firing rates, *λ_c_*, with the phase of the following four *θ-*oscillations: 1) *θ_1_* – a single 8 Hz sinusoidal wave, 2) *θ_4_* – a combination of four sinusoids, 3) *θ_M_* – a subcortical EEG signal recorded in wild-type mouse, and 4) *θ_R_* – a subcortical EEG signal recorded in a rat (**Supplemental Figure S2**; see **Methods**). The last three signals were filtered in the *θ*-domain of frequencies (6–12 Hz). The distribution of the learning times, the histograms of the mean learning times, and the histograms of the relative variability, *ξ*, for all four different theta cases are shown in **Supplemental Figures S3 and S4**.

To compare the *θ*-off and *θ*-on cases, we performed two-sample Kolmogorov-Smirnov (KS) tests for all pairwise combinations of the studied sample sets [21]. This produced a 5×5 matrix of the *p*-values, *p_ij_*, where *i*, *j* = 0 (no theta), *θ_1_*, *θ_4_*, *θ_M_*, and *θ_R_*. Black squares signify a statistically significant difference between cases *i* and *j* (*p<*0.05); gray squares signify no statistically significant difference. The statistical difference diagrams for the sets of *T_min_* values (**Supplemental Figure S3**) and for the learning time variability (**Supplemental Figure S4**) indicate that the distributions of learning times in the various *θ*-on cases were very similar, but the difference between all of these and the *θ*-off case was statistically significant.

### Theta precession makes spurious loops more transient

So far we have described the outcome of place cell ensemble activity in terms of the time at which the correct number of loops in the simplicial complex T emerges. But the learning process can also be described by how spurious loops are handled in the system. These loops are a fair representation of the subjective experience of learning. It takes time to build a framework into which new information can be properly slotted: until that framework is in place—whether it's a grasp of the layout of a neighborhood or the basic principles of a new field of study—we have incomplete hunches and many incorrect notions before experience (more learning) fills in our understanding. Translating this into topological terms, as the knowledge gaps close, the spurious loops contract. We therefore wanted to study the effects of theta precession on the dynamics of loop formation. Does a “smarter” ensemble form more spurious loops or fewer? Does it resolve those loops more quickly?

**Figure 3:**
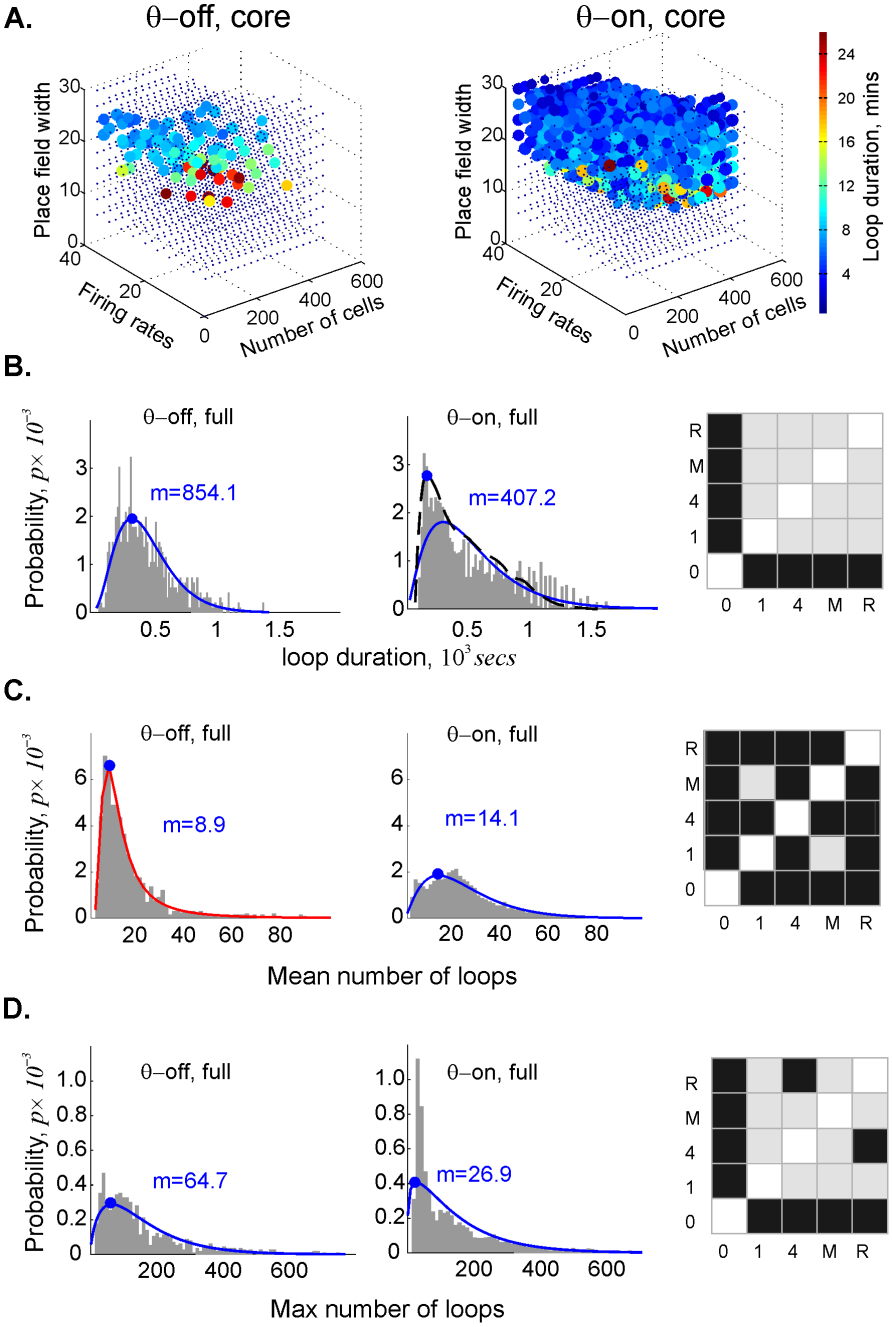
Theta phase precession reduces the duration of spurious loops in the simplicial complex. (**A**) The point clouds depict the core of the learning region, as in **Figure 2B**, for the sake of legibility. In both the *θ*-off and *θ*-on cases, note the red circles at the lower boundary of L, indicating longer-persisting loops. (**B**) Histograms showing the distributions of spurious loop durations in the *θ*-off and *θ*-on cases. We performed the Kolmogorov-Smirnov (KS) test to statistically compare the five cases against one another: 0  =  no theta, 1 = a single 8 Hz sinusoidal wave, 4 = a combination of four sinusoids, M = a subcortical EEG signal from a wild-type mouse, and R = a subcortical EEG signal recorded from a wild-type rat. Black squares indicate a significant difference (*p*<0.05) in loop duration between *θ*-off and all other cases; the *p*-values for pairwise comparisons between different *θ*-on cases reveal no significant difference (gray squares, *p*>0.2). The statistical similarity of all the *θ*-on distributions enabled us to combine the *θ*-on loop duration data to obtain better statistics for the histogram. The structure of the histograms is fit by the gamma distribution (blue lines). We used the smooth histogram profile (dashed line) to show that the typical duration of the topological loops in the *θ*-on cases is about half that of the *θ*-off case. (**C**) The typical number of loops in the *θ*-on cases is ∼40% higher than in the absence of *θ*-precession. The *θ*-off histogram is fit by the GEV distribution (red line), while the *θ*-on histogram is better fit by gamma distribution (blue). The KS diagram on the right shows that while the simulated *θ*-oscillations produce similar results, the signals recorded from rat and mouse produce statistically different distributions from the simulated signals. (**D**) The maximal number of spurious loops observed in the *θ*-on cases, fit by the gamma distributions, is less than half that in the *θ*-off case.

We concentrated on the 1*D* cycles, which represent path connectivity within the simplicial complex, because they are more numerous and thus produce more robust statistics than the 0*D* cycles. **Figure 3** shows that *θ-*precession shortened the duration of the spurious loops. The KS test reveals a statistically significant difference between the lifetimes of spurious loops in the *θ*-off case and those in all the *θ*-on cases (**Figure 3B**). To simplify the presentation of the results produced by the statistically similar *θ*-on cases, we combined the data on spurious loop duration from all four *θ*-driven maps into a single histogram. It is interesting to note that the probability distributions for loop dynamics are typically better fit by the gamma distribution (**Figure 3B–D**). In the *θ*-driven maps, a typical spurious loop persisted for 50% less time than it would without *θ-*precession (**Figure 3B**). It is worth noting that the spurious loops persisted longer at the lower boundary of the learning region, where the mean firing rates and place field sizes are smallest. This makes sense, insofar as whatever information appears will take longer to be corrected.

Statistical analysis of the largest number of loops observed at any given point over the course of the map formation period also differentiated *θ*-driven from *θ*-off maps. Curiously, *θ*-driven cases tended to produce a significantly higher mean number of spurious loops than the *θ*-off case (**Figure 3C**), but with a lower peak number of loops (**Figure 3D**). This implies that *θ*-precession enhances the speed of spatial learning overall at the price of creating more (transient) errors; lots of spurious loops are formed early on, but they disappear faster. The KS test shows that the distributions of the mean number of loops in all *θ*-on cases differ from one another; only the maps driven by the two simulated *θ-*signals gave statistically similar results.

### Too many simplices make learning less efficient

In our model, spatial learning can be quantified by the time required for the emergence of correct topological information, but it can also be quantified by studying the simplicial complex itself. We noted earlier that the structure of the simplicial complex approximates the structure of the environment. Similarly, it is possible to conceive of a simplex as a mathematical analogue to a cell assembly (a group of at least two cells that repeatedly co-fire and form a synapse onto a readout neuron), and to view the simplicial complex as analogous to the realm of possible connections within the hippocampus. We were therefore curious: since it is in the interest of neural function to be efficient, how many cell assemblies (simplices) does it take to encode a given amount of information? We would predict that the fewer the connections, the better, for the sake of efficiency.

One of the major characteristics of a simplicial complex T is the number of *n*-dimensional simplices it contains, traditionally denoted as *f_n_*. The list of all *f_n_* –values, (*f_1_*, *f_2_*, …, *f_n_*), is referred to as the *f*-vector [22]. Since the *D*-dimensional simplices in T correspond to (*D*+1)-ary connections, the number of which depends on the number of vertices, *N*, we considered the *f_n_* values normalized by the corresponding binomial coefficients, 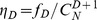
which characterize the number of simplices connecting vertices in the complex T. We can consider *η* an index of the connectivity of the simplicial complex. Since we model 2*D* spatial navigation, we analyzed the connections between two and three vertices, i.e., the 1*D* and 2*D* simplices, of T (the number of 0*D* simplexes normalized by the number of vertices in T is *η_0_*≡1).

**Figure 4:**
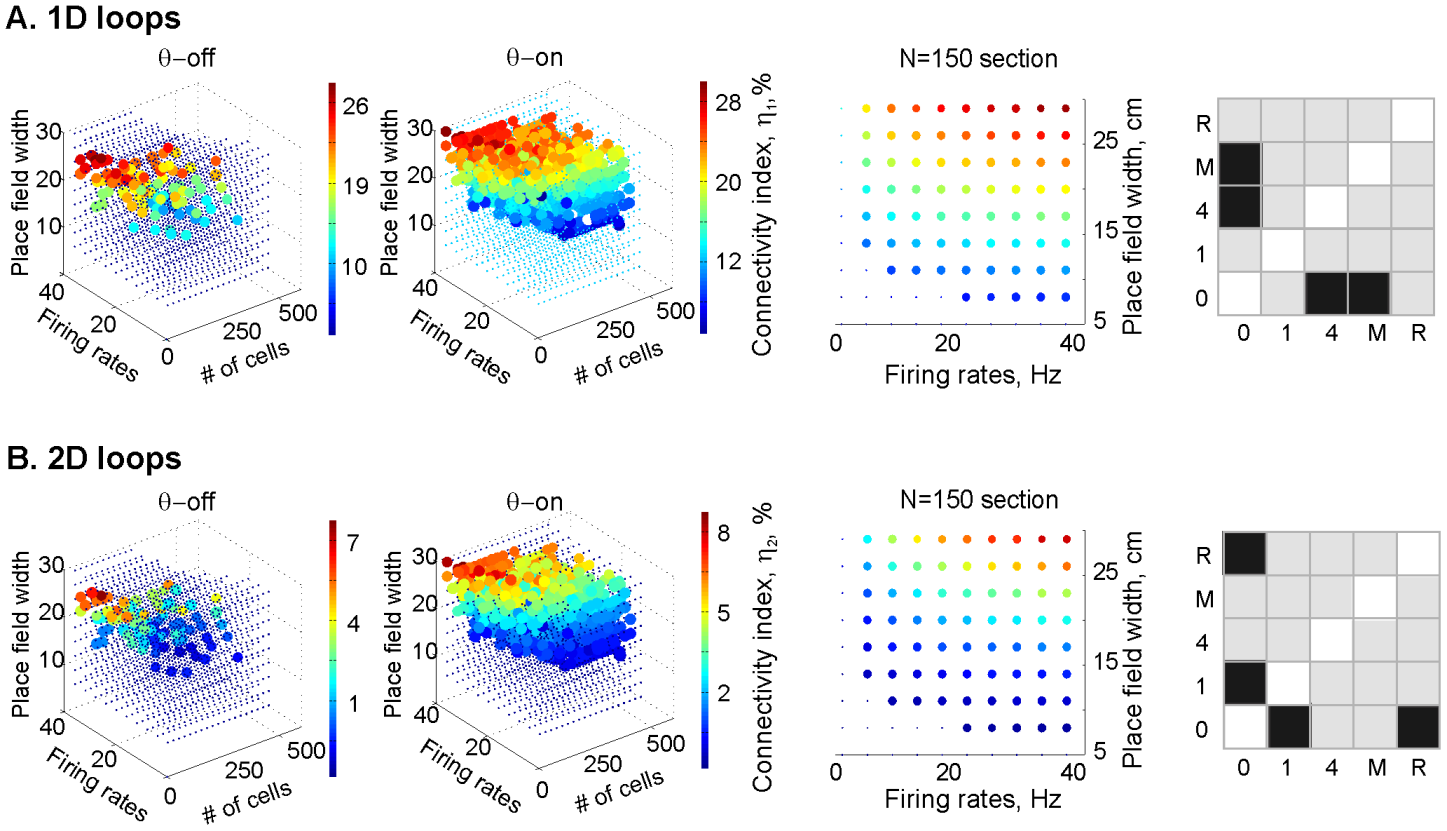
Too many simplices correlates with inefficient learning. Distributions of the (**A**) 1*D* and (**B**) 2*D* connectivity indexes, *η_1_* and the *η_2_*, aross the ensembles that form correct maps at least 70% of the time (convergence rate *ρ* ≥0.7) and have low relative variability (*ξ*<0.3, as in **Figs. 2** and **3**) in the *θ*-off and the *θ*-on cases. The distribution of the *η_1_* and the *η_2_* values over the learning region L indicates that the normalized number of 1D and 2D simplices scales with the number of combinatorially possible connections in the place cell ensemble. Correspondingly, the structure of the normalized connectivity in the temporal simplicial complexes can be seen in the cross-sections of the learning region (third column; notice that the normalized connectivity increases with a rise in both the mean ensemble firng rate and the mean ensemble place field size, in both the *1D* and *2D* cases). In the ensembles with high firing rates and low spatial selectivity, up to 25% of place cell pairs and up to 8% of place cell triplets are coactive. The KS test shows that more simplices make for inefficient learning whether or not there is theta precession, though there is a difference between the *θ*-off and the *θ*-on cases when considering the *1D* and *2D* connectivity indices together.

**Figure 4** shows the distribution of the normalized number of simplices at the time the correct signature is achieved (*η_1_* and *η_2_*, for 1*D* and 2*D*, respectively). As expected, the number of simplices was smaller at the lower boundary of the learning region L (the base of the point cloud) and increased towards the top of L where place fields are larger and the firing rates are higher, each of which would produce more place cell co-firing events. Remarkably, the number of simplices depended primarily on the mean place field size and on the mean firing rate of the ensemble, and not on the number of cells within the ensemble. This suggests a certain universality in the behavior of place cell ensembles that is independent of their population size. In the ensembles with smaller place fields and lower firing rates, about 1.5% of place cell pairs and 1.7% of the triplets were connected, and this was enough to encode the correct topological information, whereas in the ensembles with low spatial selectivity and higher firing rates, 25% of pairs and 8% of triplets were connected. These ensembles, in which the place fields and spike trains will by definition have a lot of overlap, are forced to form many more 1*D* and 2*D* simplices in order to encode the same amount of information and are thus less efficient (**Figure 4**, third column).

According to our model, such ensembles and the hippocampal networks whose activity they represent are inefficient on two counts. First, these larger, more complex temporal simplicial complexes (analogous to a larger number of coactive cell groups) will take longer to form correct topological information, if they can manage it at all. Second, a larger number of coactive place cells would hamper the training of downstream readout neurons, thereby impeding reliable encoding of spatial information. This is consistent with studies showing that the number of cells participating in a particular task decreases until it reaches an optimal number that fire at a slightly higher rate than their no-longer-participating neighbors [23].

### Window size: Defining what constitutes co-firing

Our model depends on patterns of place cell co-firing, but we had not previously explored what the optimal temporal window for defining co-activity might be. Experimental work supports the widely held assumption that the temporal unit for defining coactivity ranges between tens [24] and hundreds of milliseconds [25]–[27]. Our model, however, enables us to approach the question of optimal width for the coactivity window theoretically. Clearly, if the time window *w* is too small, then the spike trains produced by the presynaptic place cells will often “miss” one another, and the map will either require a long time to emerge or it may not be established at all. One would thus expect large values *T_min_*(*w*) for a small *w*. On the other hand, if *w* is too large, it will allow cells whose place fields are actually distant from one another to be grouped together, yielding incorrect topological information. Theta rhythm itself will have a tendency to group sequential spike trains together, but clearly there must be limits to this, or else some place cells would be read downstream as co-firing when they actually are not. Therefore, there should exist an optimal value of *w* that reliably produces a finite, biologically relevant learning time *T_min_* at which the learning region L is robust and stable.

**Figure 5:**
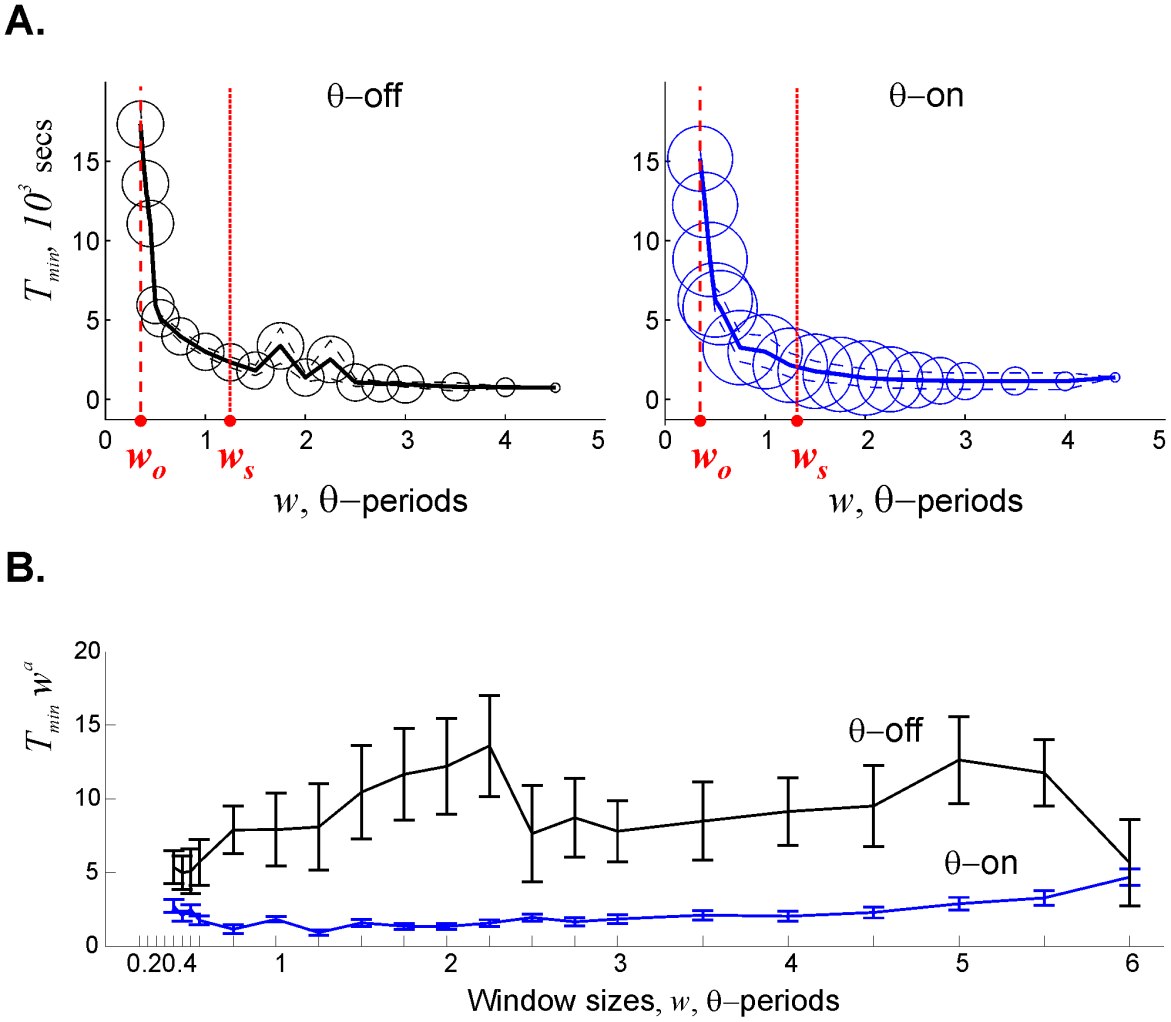
The effect of theta precession on learning time depends on window width. (**A**) Dependence of learning time, *T_min_*, on window width, *w*, for the ensemble *N* = 350, *f_max_* = 28 Hz, and *s* = 23 cm, in the *θ*-off and in the *θ*-on cases. The radius of the circles indicates the percentage of times the map converges on correct information (the larger the radius, the greater percentage of convergence). In both cases, the first convergence to the correct signature occurs as the window widens to about *w_o_* = 0.2 *θ*-periods (one *θ*-period is approximately 125 msec). At this “opening” value the learning time is about 300 mins, which is about 60 times higher than the typical value obtained for a time window of two *θ*-periods. The learning time *T_min_* at the “opening” values of *w* is highly sensitive to variations of *w*: as *w* changes from 0.2 to 0.3 *θ-*periods, the learning time *T_min_* changes by over 300%. As the integration time *w* increases, the dependence *T_min_*(*w*) rapidly drops off until it plateaus at about *w_s_*∼1.5 *θ*-periods, where it stabilizes. As *w* increases further, the learning time *T_min_* does not change significantly, but learning becomes less and less reliable, i.e., the likelihood of the spatial map converging to accurate topological information drops. Finally, the neuronal ensemble fails to encode the correct topological information at *w* ??? 4.5 *θ*-periods. (**B**) The dependence of the learning time on *w* shown above suggests that *T_min_* is inversely proportional to a power of the window width, *T_min_* = *C/w^α^*, where *α* and *C* are constants. To test this hypothesis, we selected the maps that converged for at least 19 out of 24 values of *w*, and computed the product *T_min_ w^α^* for 12 values of *α* taken from the interval 1<*α*<2 (See **Supplemental Figure 7**). The results show that the product *T_min_ w^α^* remains bounded for the entire range of window sizes. While in the *θ*-off case the variation of the product *T_min_ w^α^* remains large, the *θ*-on case it is nearly constant, which suggests that a nearly hyperbolic relationship *T_min_ w^α^* = *C* is more tight in the *θ*-on case.

We assume that the capability of a read-out neuron to detect place cell co-activity is specified by a single parameter, the width of the integration time window *w*, over which the co-appearance of the spike trains is detected. (We considered the possible effect of time bin position on co-activity, but found this did not affect outcome; see **Methods**.) We defined cell coactivity as follows: if presynaptic neurons *c_1_* and *c_2_* send a signal to a read-out neuron within a certain time window *w*, their activity will be interpreted as contemporaneous. The width of this time window may be positive (*c_2_* becomes active *w* seconds after *c_1_*) or negative (*c_2_* becomes active while *c_1_* is still firing). We studied window widths *w* for which the place cell spike trains would eventually be able to produce the correct topological signature (the Betti numbers, see **Methods** in [4]). In order to describe the dependence of learning times on the window width, *T_min_*(*w*), we scanned an array of 24 values of *w* (ranging from 0.1 to 5 *θ*-periods) for each combination of the parameters (mean *s, f*, and *N*) and noted the width of the value *w_o_*, at which the map began to produce the correct topological signature. We call this initial correct window width the “opening” value. A typical result is provided by an ensemble with *f* = 28 Hz, *s* = 23 cm and *N* = 350, in which an accurate topological map emerges at a fairly small window width, *w_o_*∼25 *msec* (**Figure 5**). The distribution of the opening window widths shows that *w_o_* may exceed 1.5 *θ-*periods (∼25 *msec*), which matches the slow *γ*-period [24], [28] (**Supplemental Figure S5**). Since at this stage *γ*-oscillations have not been explicitly built into the model, this correspondence is coincidental, if suggestive.

**Figure 6:**
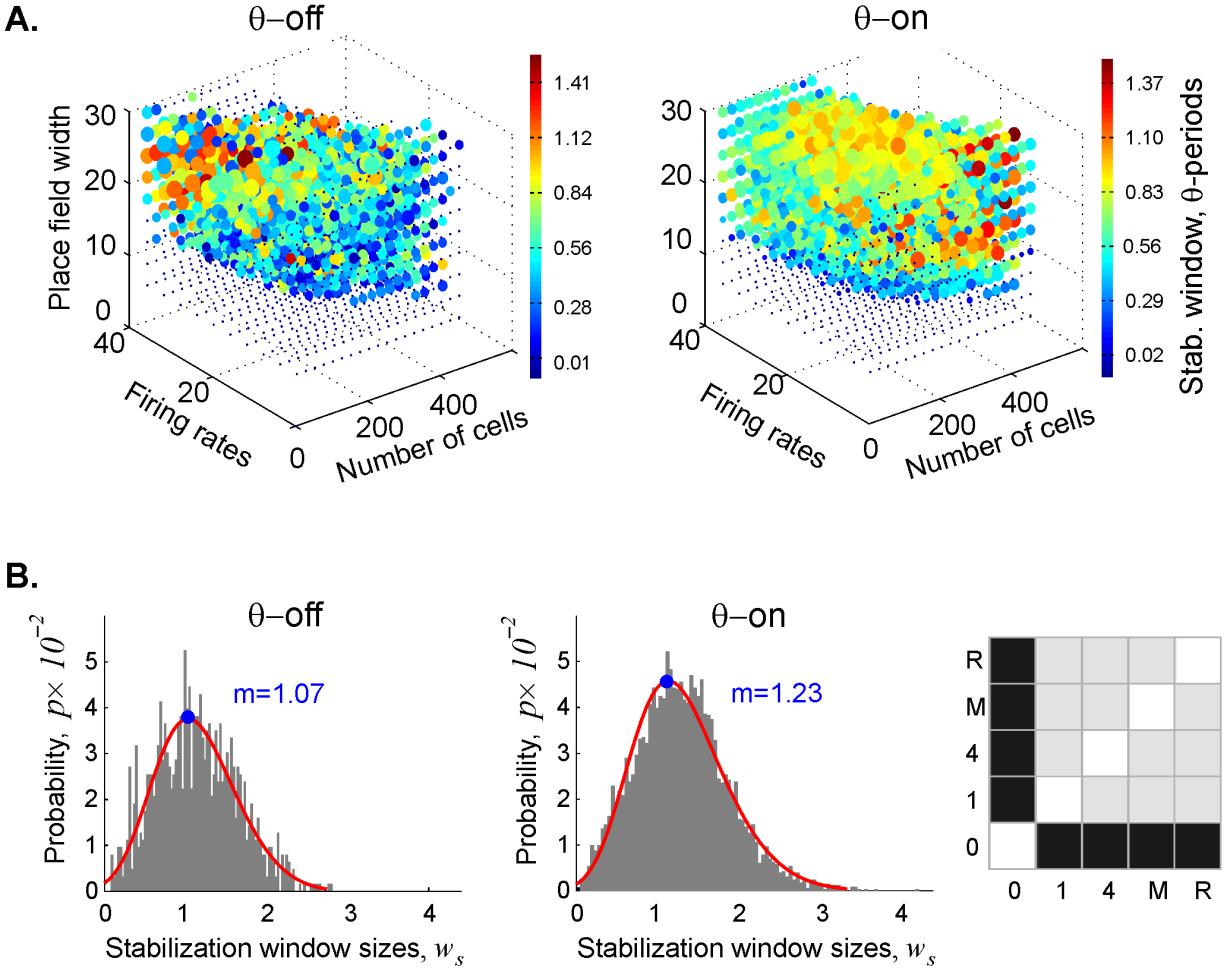
The optimal window width is slightly larger than a single *θ*-period. (A) The distribution of the stabilization window widths *w_s_* across all convergent maps (any finite *ρ* and *ξ* values) in *θ*-off and *θ*-on cases. (B) Statistical distribution of *w_s_* in *θ*-off and *θ*-on cases and show a 15% statistically significant difference in the typical value of the *w_s_* for the *θ*-on cases.

As expected, the values of learning times at *w_o_* were rather large: *T_min_*(*w_o_*) ∼20 minutes in *θ*-off case and *T_min_*(*w_o_*) ∼30 minutes in the *θ*-on case, and in some cases exceeding one hour (mostly for the ensembles with low firing rates). For small window widths, the value of the learning time *T_min_*(*w*) was very sensitive to variations in *w* (**Supplemental Figure S6**). As *w* increased, however, the learning time reached a plateau around some larger value *w_s_*. This implies that in order to produce stable values for *T_min_* that are biologically plausible, the values of the window widths should start around *w_s_*. The distribution of the *w_s_* values demonstrates that in the *θ*-off case the stabilization is typically achieved at approximately one *θ*-period, and in the *θ*-on case at about ∼1.2–1.5 *θ*-cycles **(** **Figure 6**), which justifies our choice of a two *θ-*period window width for the computations and corresponds well with the predicted limit of 150 msec for *θ*-cycle cofiring in sequence coding [29]. Further increasing the integration time window *w* did not significantly alter the learning time *T_min_* in L; instead, the rate of map convergence decreased until the maps completely fail to encode the correct topological information at *w* ζ 4.5 *θ*-periods. From the perspective of our current model, the range of optimal window widths *w* is between 20–25 msec and 0.5 secs.

Finally, we sought to uncover a relationship between learning time and window width. Our analysis suggests that *T_min_* is inversely proportional to a power of the window width **(** **Figure 5B**, **Supplemental Figure S7**).

## Discussion

We have used methods derived from persistent homology theory and algebraic topology to simulate one way physiological data from individual neurons might combine at the level of a neuronal ensemble to enable spatial learning [4], [6], [16]). With this model, we found that *θ* phase precession significantly increased the size of the learning region L, reduced the learning time *T_min_*, and changed the dynamics of spurious loop formation. Together, these findings provide compelling support to the notion that phase precession enhances spatial learning. Given that this has been speculated to be one purpose of *θ* phase precession [17]–[19], the results of the present study validate our spatial learning model. The correspondence of the model's optimal window width with values obtained from animal experiments further underscores the physiological relevance and predictive power of the model.

### *θ* phase precession, learning, and the importance of levels of scale

Numerous experiments have demonstrated that *θ* precession is important for spatial learning. *θ-*power increases with memory load during both spatial and non-spatial tasks in humans [30], [31] and in rodents [32], [33]; spatial deficits correlate with a decrease in the power of theta oscillations in Alzheimer's disease [34] and in epilepsy [35], [36]. If *θ-*signal is blocked by lesioning the medial septum (which does not affect hippocampal place cell representations), it severely impairs memory [37] and the acquisition of new spatial information [38]. Recent experiments demonstrate more directly that destroying *θ* precession by administering cannabinoids to rats correlates with behavioral and spatial learning deficits [17], [39]. But at what level, and through what mechanisms, does *θ* precession exert its influence? The effect of *θ-*precession on the structure of the spike trains is rather complex [40]. On the one hand, it groups cell spikes closer together in time and enforces specific sequences of cell firing, which is typically interpreted as increasing the temporal coherence of place cell activity [41]–[43]. One might predict that grouping spikes together would (somehow) speed up learning. On the other hand, *θ-*precession imposes extra conditions on the spike timing that depend on *θ-*phase and on the rat's location with respect to the center of the place field through which it is presently moving. Since every neuron precesses independently, one could just as well predict that *θ* modulation would either restrict or enlarge the pool of coactivity events, which in turn would slow down learning at the level of the downstream networks, and that the beneficial effect of the *θ* rhythm is a higher-order phenomenon that occurs elsewhere in the brain.

Our results suggest that *θ* precession may not just correlate with, but actually be a mechanism for, enhancing spatial learning and memory. The interplay of *θ* precession and window width, especially the extremely long learning times at the opening window width *w_o_*, is particularly illuminating here. As noted, theta precession acts at both the ensemble and the individual neuron level: it groups spikes together, but each neuron precesses independently. When the time window is sufficiently wide, the coactivity events are reliably captured, the first effect dominates, and the main outcome of theta precession is to supply grouped spikes to downstream neurons. For very small time windows, however, the system struggles to capture events as coactive, and the extra condition imposed by phase precession acts as an impediment: detected coactivities are rarer, and learning slows down. Put more simply, imagine in **Suppl. Figure S8** that the window is only one spike wide: in a train of 10 spikes that overlaps by one spike with another train, it will take 10 windows before the overlap is detected.

It is noteworthy that the presence of theta precession was clearly more important than the details of the oscillation. Although theta precession enhanced learning in our simulations, learning times were relatively insensitive to the details of the theta precession chosen. One might expect differences in spike train structure induced by the four different *θ*-signals studied to alter the dynamics of the persistent loops and thus learning efficiency. Our results show, however, that differences that would matter at the level of individual cells are averaged out at the level of a large ensemble of cells.

Here again the model shows its particular strength: it allows us to correlate parameters of activity at the level of individual neurons with the outcome at the level of an ensemble of hundreds of cells, providing a framework for understanding how micro-level changes play out at the behavioral level. Interestingly, we also saw a difference between the micro and macro levels when we considered whether the placement of a temporal window affected what would be considered co-activity (see **Methods** and **Supplementary Figure S8**). In theory it should, but the effect at the macro level washes out and we found that only the temporal width of the window matters for learning time.

### Simplicial complex formation as a metaphor for learning

Beyond validating the model as a reliable way to study physiological aspects of spatial learning, we have gone further in this work to analyze the simplicial complex itself as a way of describing learning. As a rat starts to explore an environment, some cells begin to form place fields. Then, the co-firing of two or more place cells will define the respective places as connected in space and temporal experience and will create corresponding simplices in the simplicial complex T. With time, these simplices form a chain corresponding to the animal's route through the space. If the environment is bounded, the rat will discover new connections between the same places (arriving to the same location via different routes). As a result, the chains of connected simplices grow together to form loops. Existing loops become thicker and may eventually “close up” and disappear, yielding surfaces. The appearance of such surfaces is significant: the closing up of a *D*-dimensional surface corresponds to the contraction, or disappearance, of one of its boundaries, which itself is a *D*1-dimensional loop. Eventually, the structure of the simplicial complex saturates such that no new simplices (connections between places) are produced and no more loops contract because all that could close have already closed. At this point, the saturated simplicial complex T encodes not only the possible locations of the rat, but also connections between the locations, along with the information about how these connections can be deformed, e.g., whether they are contractible or whether they encircle an obstacle and cannot be contracted into a point. Thus, the saturated temporal simplicial complex T is a framework that unifies information about places and spatial-temporal relationships between them. This framework might correspond fairly well to the structure formed by synaptic connectivity in the rat's hippocampus, which allows the rat to explore and retrieve information by “pinging” the network, without physical navigation [44].

In addition to the practical benefits of a model that consistently produces biologically relevant results, there is a special appeal in the ability of algebraic topology to provide insight into the mechanisms of learning. It is fitting that a method developed to simplify the analysis of high-dimensional data with many coordinates might itself represent how the brain approaches a similar challenge in the real world.

## Materials and Methods

### Ensemble parameters

An ensemble of *N* cells is described by *N* peak rates, *f_1_, f_2_, …, f_N_*, and *N* place field widths, *s_1_*, *s_2_*, …, *s_N_*. As in [4], we assume that the values *f_i_*, and *s_i_*, are log-normally distributed around a certain ensemble-mean firing rate and the ensemble-mean place field size, with the variances *σ_f_* and *σ_s_* respectively. To simplify the analysis, we assume that the variance of the firing rate, *σ_f_*, and of the place field size, *σ_s_*, are proportional to their means, *σ_f_ =  af* and *σ_s_ = bs*, so that the distribution of the firing rates and of the place field sizes are defined by a single parameter, *f* and *s* respectively. The protocol of the simulations was similar to [4]: the trajectory was fixed, but the place field centers, *r_c_*, are randomly scattered in the environment for each simulation.

### Theta signals

The first simulated *θ-*signal (*θ_1_*) contained a single sinusoidal oscillation with the frequency *f* = 8.0 Hz. The second simulated *θ-*signal (*θ_4_*) was obtained by combining four sinusoids, 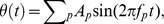
with frequencies *f_1_* = 6.5 Hz, *f_2_*, = 8.65 Hz, *f_3_* = 10.0 Hz, and *f_4_* = 11.5 Hz, filtered between 6 and 12 Hz. The third and the fourth *θ-*signals (*θ_M_* and *θ_R_*) were obtained by filtering subcortical EEG signals recorded in mice and in rat, filtered between 6 and 12 Hz.

### Theta precession

As the rat just enters the place field of a cell *c_i_*, that cell prefers spiking at a high *θ*-phase, φ_*_∼2π, and as the animal moves over a distance *l* towards the center, the preferred phase decreases, reaching φ_*_∼0 as the rat exits the place field [10], [45]. We approximate this dependence by a linear function, 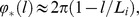
where *L_i_* is the size of the place field *c_i_*, *L_i_*∼3*s_i_*, To simulate the coupling between the firing rate and the *θ-*phase, we modulate the original Gaussian firing rate by a phase-dependent factor Λ(*φ*), which peaks around 0,
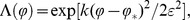
where the width *ε* we defined as the ratio of the mean distance that rat travels during one *θ-*cycle to the size of the place field, *ε  = v/Lf*. Typical examples of the resulting theta precession of spiking are shown in **Supplemental Figure S2**. For persistence methods see [4].

### Choice of environment

We conducted our simulations in small environments (1×1 m). In the **“Reducing computational cost by subdividing maps”** section (see also **Supplementary Figure S9**) we show that direct computations based on the Mayer-Vietoris theorem [14] demonstrate that the spatial map corresponding to a larger environment can be split in several smaller pieces, and that the connectivity of the entire spatial map can be computed piece-by-piece, so that the total learning time is approximately equal to the times required to “learn” its parts. This observation helps to reduce the computational cost of the algorithm. In addition, simulating the maps in smaller environments allows us to avoid multiply connected, “patchy” place fields (in smaller environments there is a lesser chance of observing more than one component of a place field, as occasionally happens when a place cell fires in more than one place) and helps to bring the density of place cells closer to experimental values.

### Additional temporal coactivity parameters

Finally, we notice that, given a particular integration time window width *w*, the co-activity between two specific spike trains may or may not be detected depending on the position of the time bins (**Suppl. Figure S5**). We studied this effect by shifting the time windows 10 times over 10% of the fill window width *w* and recomputing *T_min_*, and we saw no difference in the outcome. This implies that *T_min_* does not depend on the shift of the time window: over the typical learning time scale, the effect produced by the bin shifts averages out. Second, it is clear that a fixed time width is not physiological, because a realistic window size will “jitter” from cell to cell and from moment to moment. However, a direct simulation conducted for *w* equal to two *θ-*periods shows that adding up to 50% “jitter” to the window width does not affect the learning times: *T_min_* remains virtually invariant with respect to the amplitude of the bin size noise. This allows us to simplify the computations by using a single parameter *w* to characterize coactivity.

### Reducing computational cost by subdividing maps

One of the major computational difficulties in simulating hippocampal spatial maps is the time it takes to analyze a large number of simplices: in our original paper [4], simulating the map formation time for each set of parameters (each variation tested 10,000 times) took several months for a 2×2 m virtual space. Considered in mathematical terms: given *N* vertexes in the simplicial complex T, the number of 1*D* links scales as ∼*N^2^*, and the number of 2*D* facets scales as ∼*N^3^*. The topological relationships between them are then defined by a∼*N^2^*× *N^3^* incidence matrix [46]. Due to restrictions in computational power, we can investigate ensembles that include up to 400 cells, but in the actual hippocampus there are on the order of 4000 cells active in a given experimental environment [47], [48].

To reduce the computational load, we took advantage of the Meyer-Vietoris theorem, which allows us to simulate the map by breaking it into its constituent parts. Using Meyer-Vietoris, it is possible to compute the homological characteristics of the entire space *X* from the homological characteristics of its constituent parts [46]. Specifically, if a space *X* is split into pieces *A* and *B*, *X* = *A*∪*B*, then the homologies of *X*, *H_q_*(*X*), are related to the homologies of its parts, *H_q_*(*A*) and *H_q_*(*B*), via the so-called long exact sequence:
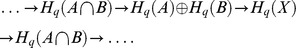
If the overlap, *A*∩*B*, is topologically trivial, *H_q_*(*A*∩*B*)* = 0*, then the sequence reduces to just
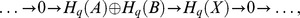
in which case the exactness of homomorphisms implies that
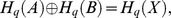
i.e., the homologies of the whole space are equal to the direct sum of the homologies of its parts. As a consequence, the Betti numbers from both *A* and *B* can be combined to accurately provide topological information about the whole space *X*.

This observation can be used to divide the map formation times of the temporal simplicial complex T(*T*). If our virtual rat spends time *T_A_* and *T_B_* in part *A* and in part *B*, respectively, then assuming that *A* and *B* meet but do not overlap, the total map formation time for the whole environment, *T_X_*, can be estimated as
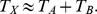



A more complete discussion of the mathematical aspects of this dividing approach will be given elsewhere; for example, there is a scale at which space can be no further atomized, and this will require considerable effort to define the size of these ‘atoms’ and account for these size limits. (At present, it appears that an atom of space is approximately the size of two to three place fields.) Here we present some numerical results justifying the piecewise computations (**Supplemental Figure S9**). We simulated the rat's movement in a large 2×2 m environment with two holes, which we formally divided into 2, 3 or 4 parts. After the fragments have been chosen, we counted the time spent in each region, and, by using only the spikes fired within a given region computed each region's own learning times. **Supplemental Figure S9** shows that the sum of these times is similar to total time spent by rat in the entire arena (the differences are not statistically significant). The second scenario is illustrated in the figure below.

### Place cell learning dynamics

Using the adaptive filtering method in [49] it was estimated that in novel environments, place fields form in about four minutes, starting at the background stochastic spiking level of 0.1 Hz [15], [50], [51] and gain spatial specificity in about the same amount of time. After that, place cells begin to (co)fire in a place-specific manner, encoding spatial locations [52]. To include the effect of place field formation into the model, we simulated place cell ensembles with time-dependent firing rate amplitudes, *f_i_*, and time-dependent place field widths, *s_i_*, defined as 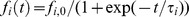
and 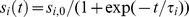
in which *τ_i_* defines the slope of the sigmoid. We chose the typical *τ_i_*-value, *τ_mean_*, equal to 240 seconds (see **Supplemental Figure S10**) and the starting points start at 0. The results shown in **Supplemental Figure S10** demonstrate that the place field formation produces only an additive effect on the overall spatial map formation time: the average map formation time increases by 120% of the *τ_mean_* with respect to the “base” value obtained for the stable place cells.

## Supporting Information

Figure S1 **Theta precession enhances learning across all ensembles that can converge onto the proper topological information.** (**A**) Theta precession increases convergence rates across all ensembles in the learning region L. (**B**) The corresponding probability distributions for *θ*-off and *θ*-on (left and right, respectively) show that theta precession greatly increases the probability of proper map formation. The KS test shows this difference is statistically significant.
(TIF)

Figure S2 **The four theta signals used to drive place cell ensembles.** (**A**) Theta oscillations tested: “1 sinusoid”  =  single frequency *f* = 8.0 Hz oscillation; “4 sinusoids”  =  superposition of four harmonics with frequencies *f_1_* = 5.2 Hz, *f_2_* = 6.5 Hz, *f_3_* = 8.65 Hz, *f_4_* = 10.0 Hz and *f_5_* = 11.5 Hz, filtered between 6 and 12 Hz; “mouse”  =  wild type mouse's subcortical EEG signal, filtered between 6 and 12 Hz, recorded at 10,000 Hz; “rat” =  rat subcortical EEG signal, filtered between 6 and 12 Hz, recorded at 1500 Hz. (**B**) Typical examples of phase precession diagrams for each of the four cases in (**A**). (**C**) The histograms of the instant frequencies for the four theta signals recorded at the times of spiking (at least one cell in the ensemble fires), which show clear structural difference between each of the four signals.
(TIF)

Figure S3 **Theta precession enhances learning regardless of specific theta rhythm.** (A) Theta precession enlarges the learning regardless of the specific theta rhythm. Point clouds show the minimal map formation times *T_min_* (color coded) computed for the *θ*-off case and the four *θ*-on cases: a single sinusoidal oscillation, *θ_1_*, a combination of four sinusoids *θ_4_*, subcortical EEG signals recorded from a mouse, *θ_M_*, and from a rat, *θ_R_*. Each dot corresponds to a place cell ensemble, with a specific number of place cells, *N*, the mean ensemble firing rate, *f*, the mean ensemble place field size, *s*. In all cases, we selected only the maps that converged at least 7 out of 10 times, and for which the variance of *T_min_*'s did not exceed 30% of the mean value. In the pairwise Kolmogorov-Smirnov (KS) test computed for the minimal time distributions, black squares indicate statistical significance (*p<*0.05) and gray squares indicate no significant difference. *θ*-off differs from each of the four *θ*-on cases (*θ_1_*, *θ_4_*, *θ_M_* and *θ_R_*). (B) Theta precession reduces mean learning time, regardless of specific theta rhythm. The histograms of the minimal times obtained in the *θ*-off and the four *θ*-on cases, fit by the GEV distribution, correspond to the cases shown in panel (A). The blue dot marks the position of the mode of the distribution, the corresponding value given by the number in the center of each panel. For the stable maps (the ones that converge in at least 70% of cases) the typical learning time *T_min_* in the *θ*-on cases is about 6.5 minutes, whereas in the *θ*-off case it is 20% higher. The KS test reveals that there is a statistically significant difference only between *θ*-off and any given *θ*-on case; the additional non-significant *p* values serve to emphasize how similar the *θ*-on cases actually are.Figure S4
(TIF)

Figure S4 ***θ*** **phase precession reduces the variability of the learning times, regardless of specific theta rhythm.** The histograms show that the typical value of the relative variation ξ =  Δ*T_min_*/*T_min_* in the *θ*-on cases is less than half that of the *θ*-off case, i.e., that repeated simulations of the *θ*-driven maps more reliably reproduce similar learning time values. Theta thus increases the reliability and efficiency of map formation. Interestingly, the KS test indicates that the experimentally derived rhythms (from mouse and rat) are statistically significantly different from the simulated oscillations, but in the case of the rat signal, it is possible that this is because the signal itself had a wider distribution of frequencies centered around 8 Hz.
(TIF)

Figure S5 **The opening window width is close to the** ***γ*** **-period scale.** (**A**) The cloud of the opening window width values, *w_o_*, in the *θ*-off and the *θ*-on cases for all convergent maps (any finite *ρ* and *ξ* values) indicates that at the core of the learning region, the width of the opening window sizes is similar to the slow *γ*-period scale (∼25 msec). (**B**) Combined histogram of the distributions of the opening window sizes, *w_o_*, in the *θ*-off and the *θ*-on case. All *θ*-on cases are significantly different from the *θ*-off case.
(TIF)

Figure S6 **Learning times are very large at opening window width values.** (**A**) The distribution of the mean amount of time required for the correct spatial information to converge at the opening window width, *w_o_*, across the learning region in the *θ*-off and the *θ*-on cases. (**B**) The statistical distribution of the *T_min_*(*w_o_*) 's shows that the typical learning time is about 20 minutes in the *θ*-off case vs. 30 minutes in the *θ*-on case, which is a statistically significant difference for all thetas tested in comparison with no theta.
(TIF)

Figure S7 **Learning time is inversely proportional to a power of the window width.** The test of the hypothesized inverse proportionality dependence between the learning time, *T_min_*, and the window width, *w*, *T_min_* = *C/w^α^*, where *α* and *C* are constants (see **Figure 5**). To test this hypothesis, we selected the maps that converged for at least 19 out of 24 values of *w*, and computed the product *T_min_ w^α^* for 12 values of *α* taken from the interval 1<*α*<2. The results show that the product *T_min_ w^α^* remains bounded for the entire range of *α* values. While in the *θ*-off case (colormap “cool”) the variation of the product *T_min_ w^α^* remains large, in the *θ*-on case (colormap “hot”) it is nearly constant, which suggests that a nearly hyperbolic relationship *T_min_ w^α^* = *C* holds in both cases. The smallest variation is achieved for *α* = 1.4 in the *θ*-on case and *α* = 1.28 in the *θ*-off case. Notice that as *α* varies between 1 and 2, the arms of the curve flip so the “most horizontal” position is achieved for 1<*α* ^Δ^<2.
(TIF)

Figure S8 **Window widths define what counts as co-activity.** While overlap patterns **A** and C do not depend on the position of the time bins, activity of cells in close temporal succession might, in theory, be interpreted as coactivity or not depending on the position of the time bins and/or the size of the window width (**B**). In our model, however, we find that window width is the sole determinant of co-firing at the level of neuronal ensemble activity. See **Methods**.
(TIF)

Figure S9 **The Mayer-Vietoris Theorem allows us to divide maps into sections to reduce computational cost.** **A**. An area with two holes is divided into two, three or four pieces. We selected the maps that converged to correct topological information in the full arena *and* in each one of the pieces in which the arena is split, in at least seven out of ten repetitions. **B**. We then computed the mean learning times *T_min_* in the full arena (shown by the black bars) and the divided learning times in each piece, *T_p,min_*, represented by colored bars. Colors on the bars correspond to the region colors. There was no statistically significant difference between the bars.
(TIF)

Figure S10 **Place field formation time adds to the learning time.** **A**. Differences in learning times in the ensembles with static place fields versus dynamic place fields. Red line marks the mean difference, approximately 5 minutes, which is 120% of the place field formation *τ*. (Experimentally derived place field formation takes approximately 4 minutes.) **B**. The distribution of the differences shown in panel **A**, fit with normal distribution (red) with the mean *μ* = 5.58 and variance *σ* = 6.83, and with the GEV distribution (blue) with mean *μ* = 5.66, variance *σ* = 7.88.
(TIF)
