# Supplementary figures and images for: The Effects of Theta Precession on Spatial Learning and Simplicial Complex Dynamics in a Topological Model of the Hippocampal Spatial Map
Source: PLoS Comput Biol. 2014 Jun 19;10(6):e1003651. doi: 10.1371/journal.pcbi.1003651 (PMC4063672; doi:10.1371/journal.pcbi.1003651)

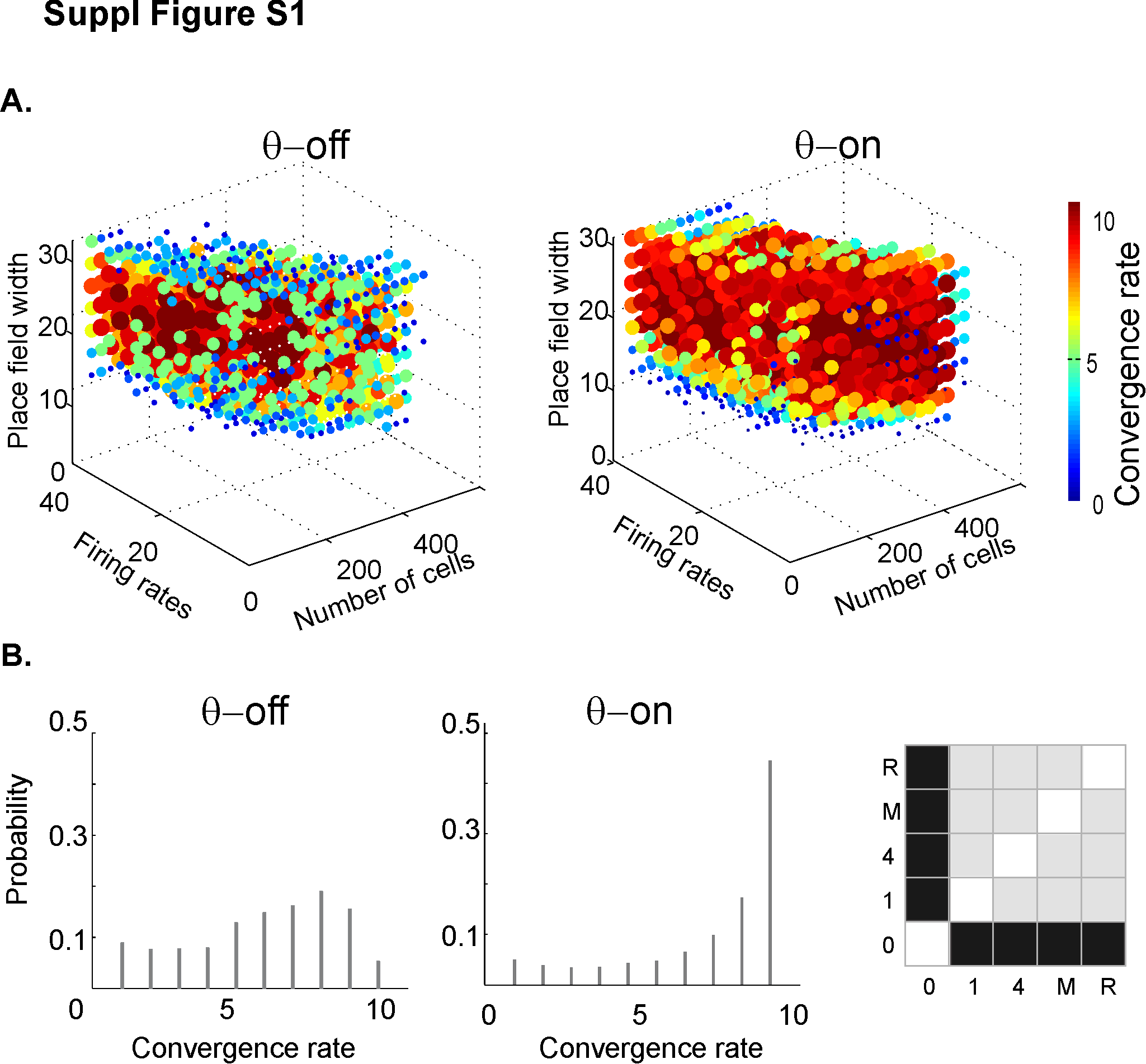

Supplement: Figure S1 — Theta precession enhances learning across all ensembles that can converge onto the proper topological information. (A) Theta precession increases convergence rates across all ensembles in the learning region L. (B) The corresponding probability distributions for θ-off and θ-on (left and right, respectively) show that theta precession greatly increases the probability of proper map formation. The KS test shows this difference is statistically significant. (TIF) [file pcbi.1003651.s001.tif]

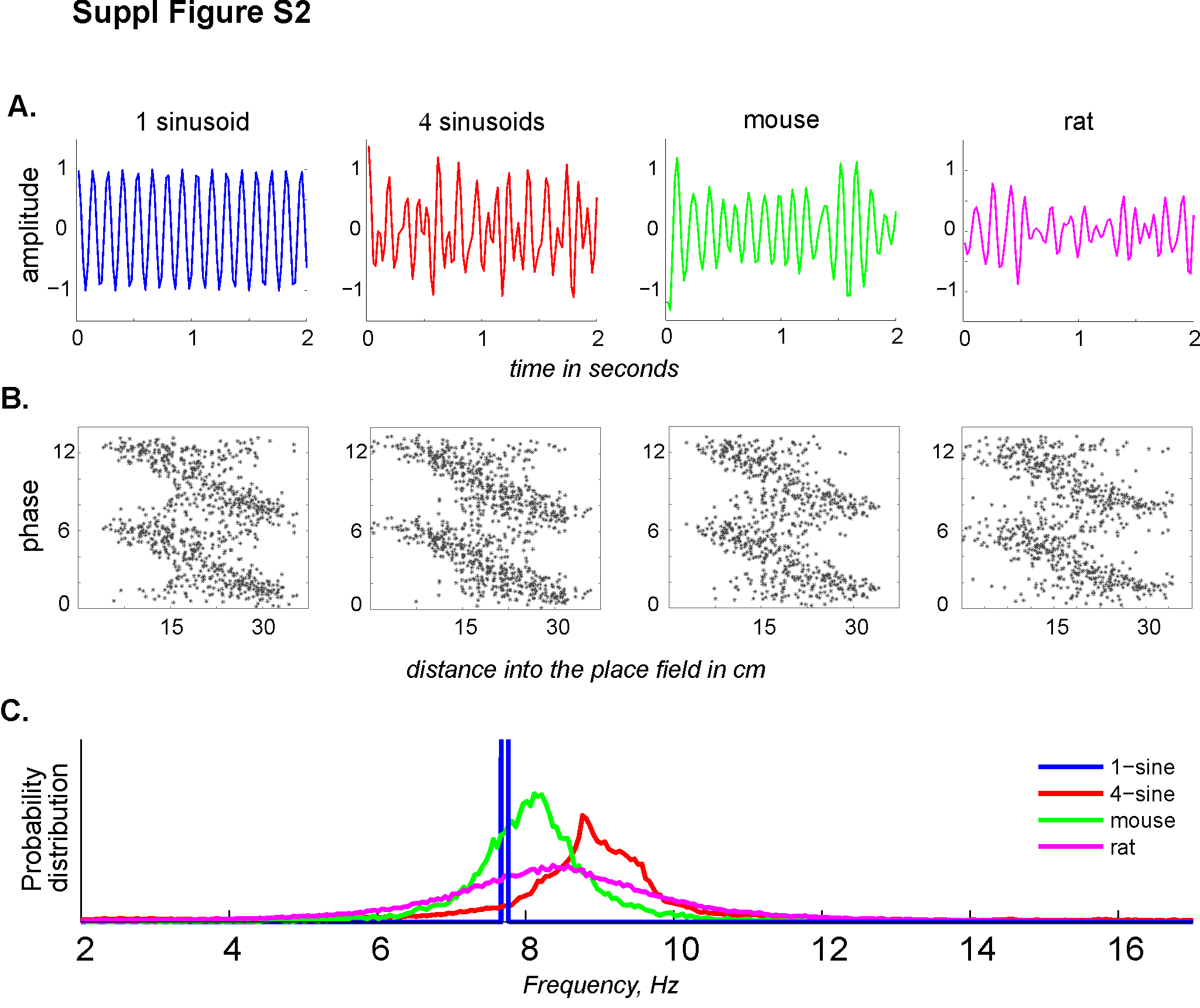

Supplement: Figure S2 — The four theta signals used to drive place cell ensembles. (A) Theta oscillations tested: “1 sinusoid” = single frequency f = 8.0 Hz oscillation; “4 sinusoids” = superposition of four harmonics with frequencies f1 = 5.2 Hz, f2 = 6.5 Hz, f3 = 8.65 Hz, f4 = 10.0 Hz and f5 = 11.5 Hz, filtered between 6 and 12 Hz; “mouse” = wild type mouse's subcortical EEG signal, filtered between 6 and 12 Hz, recorded at 10,000 Hz; “rat” = rat subcortical EEG signal, filtered between 6 and 12 Hz, recorded at 1500 Hz. (B) Typical examples of phase precession diagrams for each of the four cases in (A). (C) The histograms of the instant frequencies for the four theta signals recorded at the times of spiking (at least one cell in the ensemble fires), which show clear structural difference between each of the four signals. (TIF) [file pcbi.1003651.s002.tif]

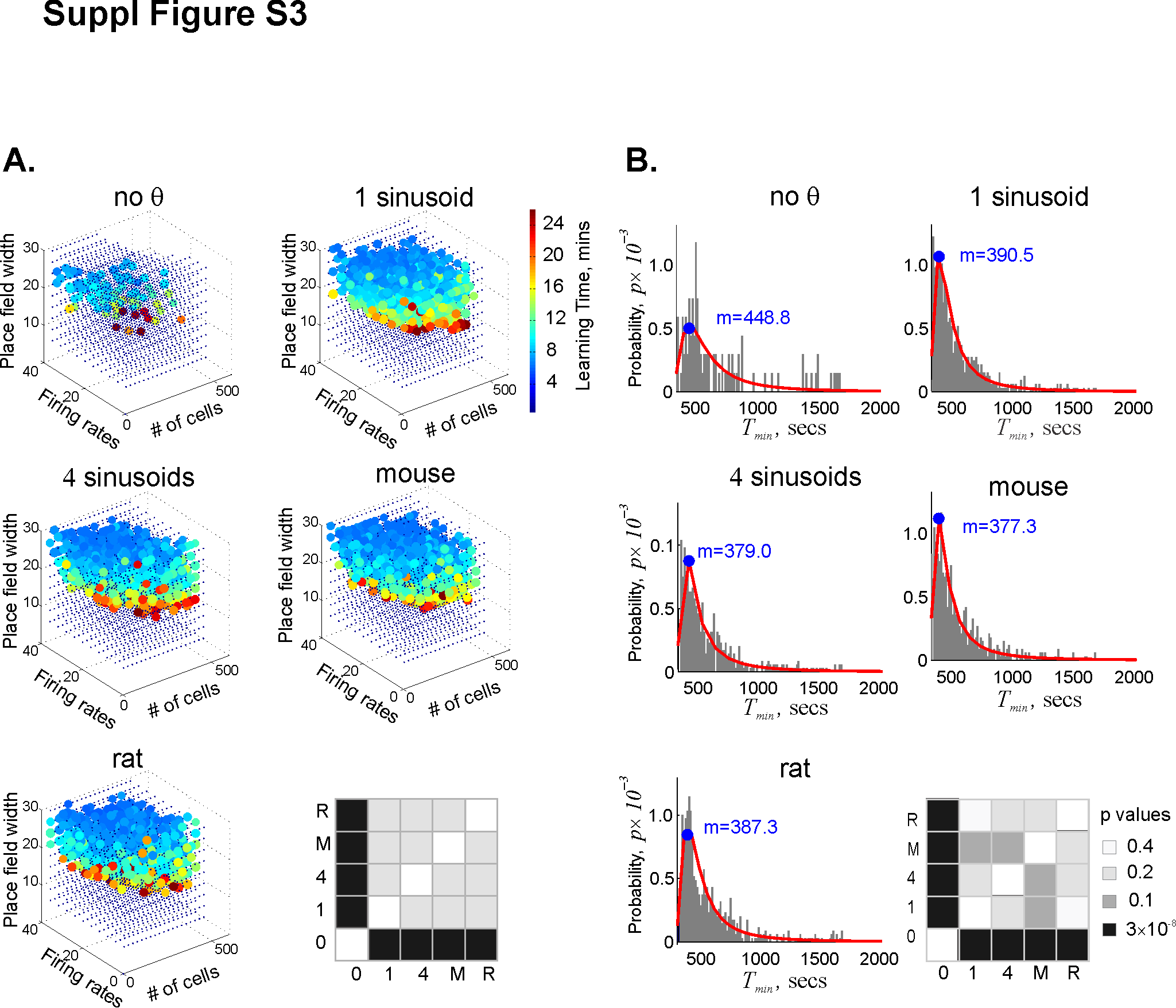

Supplement: Figure S3 — Theta precession enhances learning regardless of specific theta rhythm. (A) Theta precession enlarges the learning regardless of the specific theta rhythm. Point clouds show the minimal map formation times Tmin (color coded) computed for the θ-off case and the four θ-on cases: a single sinusoidal oscillation, θ1, a combination of four sinusoids θ4, subcortical EEG signals recorded from a mouse, θM, and from a rat, θR. Each dot corresponds to a place cell ensemble, with a specific number of place cells, N, the mean ensemble firing rate, f, the mean ensemble place field size, s. In all cases, we selected only the maps that converged at least 7 out of 10 times, and for which the variance of Tmin's did not exceed 30% of the mean value. In the pairwise Kolmogorov-Smirnov (KS) test computed for the minimal time distributions, black squares indicate statistical significance (p<0.05) and gray squares indicate no significant difference. θ-off differs from each of the four θ-on cases (θ1, θ4, θM and θR). (B) Theta precession reduces mean learning time, regardless of specific theta rhythm. The histograms of the minimal times obtained in the θ-off and the four θ-on cases, fit by the GEV distribution, correspond to the cases shown in panel (A). The blue dot marks the position of the mode of the distribution, the corresponding value given by the number in the center of each panel. For the stable maps (the ones that converge in at least 70% of cases) the typical learning time Tmin in the θ-on cases is about 6.5 minutes, whereas in the θ-off case it is 20% higher. The KS test reveals that there is a statistically significant difference only between θ-off and any given θ-on case; the additional non-significant p values serve to emphasize how similar the θ-on cases actually are.Figure S4 (TIF) [file pcbi.1003651.s003.tif]

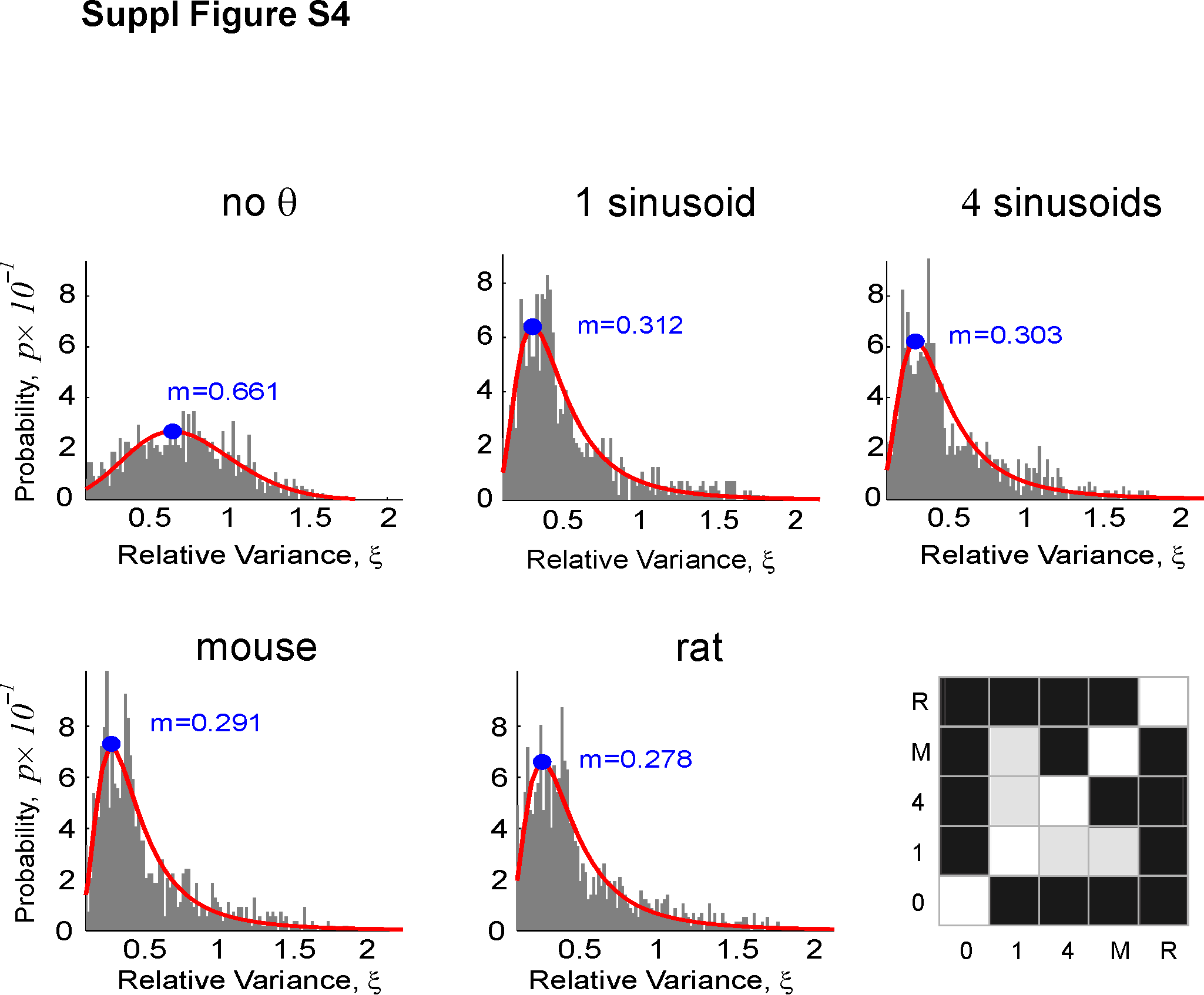

Supplement: Figure S4 — θ phase precession reduces the variability of the learning times, regardless of specific theta rhythm. The histograms show that the typical value of the relative variation ξ = ΔTmin/Tmin in the θ-on cases is less than half that of the θ-off case, i.e., that repeated simulations of the θ-driven maps more reliably reproduce similar learning time values. Theta thus increases the reliability and efficiency of map formation. Interestingly, the KS test indicates that the experimentally derived rhythms (from mouse and rat) are statistically significantly different from the simulated oscillations, but in the case of the rat signal, it is possible that this is because the signal itself had a wider distribution of frequencies centered around 8 Hz. (TIF) [file pcbi.1003651.s004.tif]

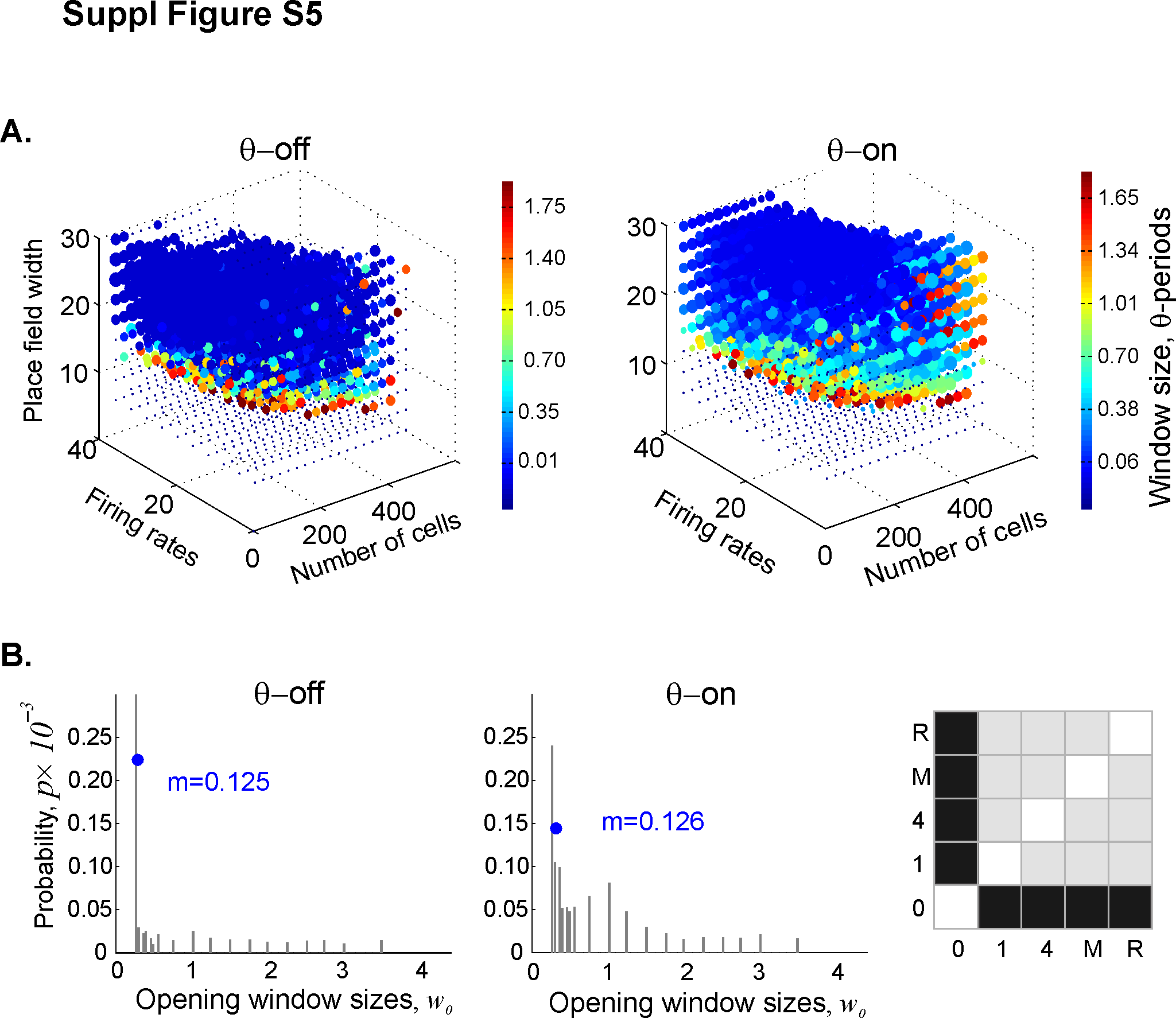

Supplement: Figure S5 — The opening window width is close to the γ -period scale. (A) The cloud of the opening window width values, wo, in the θ-off and the θ-on cases for all convergent maps (any finite ρ and ξ values) indicates that at the core of the learning region, the width of the opening window sizes is similar to the slow γ-period scale (∼25 msec). (B) Combined histogram of the distributions of the opening window sizes, wo, in the θ-off and the θ-on case. All θ-on cases are significantly different from the θ-off case. (TIF) [file pcbi.1003651.s005.tif]

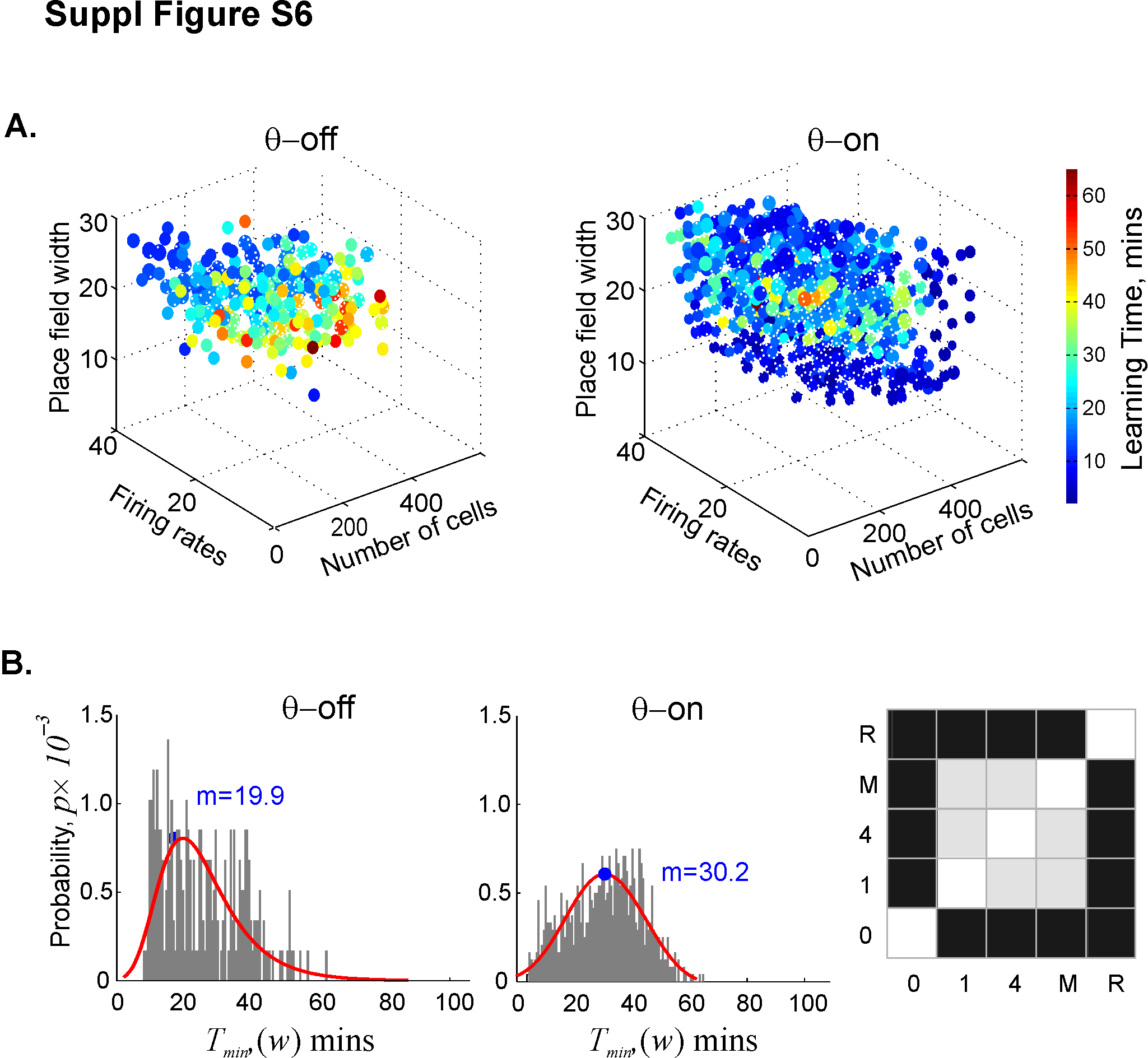

Supplement: Figure S6 — Learning times are very large at opening window width values. (A) The distribution of the mean amount of time required for the correct spatial information to converge at the opening window width, wo, across the learning region in the θ-off and the θ-on cases. (B) The statistical distribution of the Tmin(wo) 's shows that the typical learning time is about 20 minutes in the θ-off case vs. 30 minutes in the θ-on case, which is a statistically significant difference for all thetas tested in comparison with no theta. (TIF) [file pcbi.1003651.s006.tif]

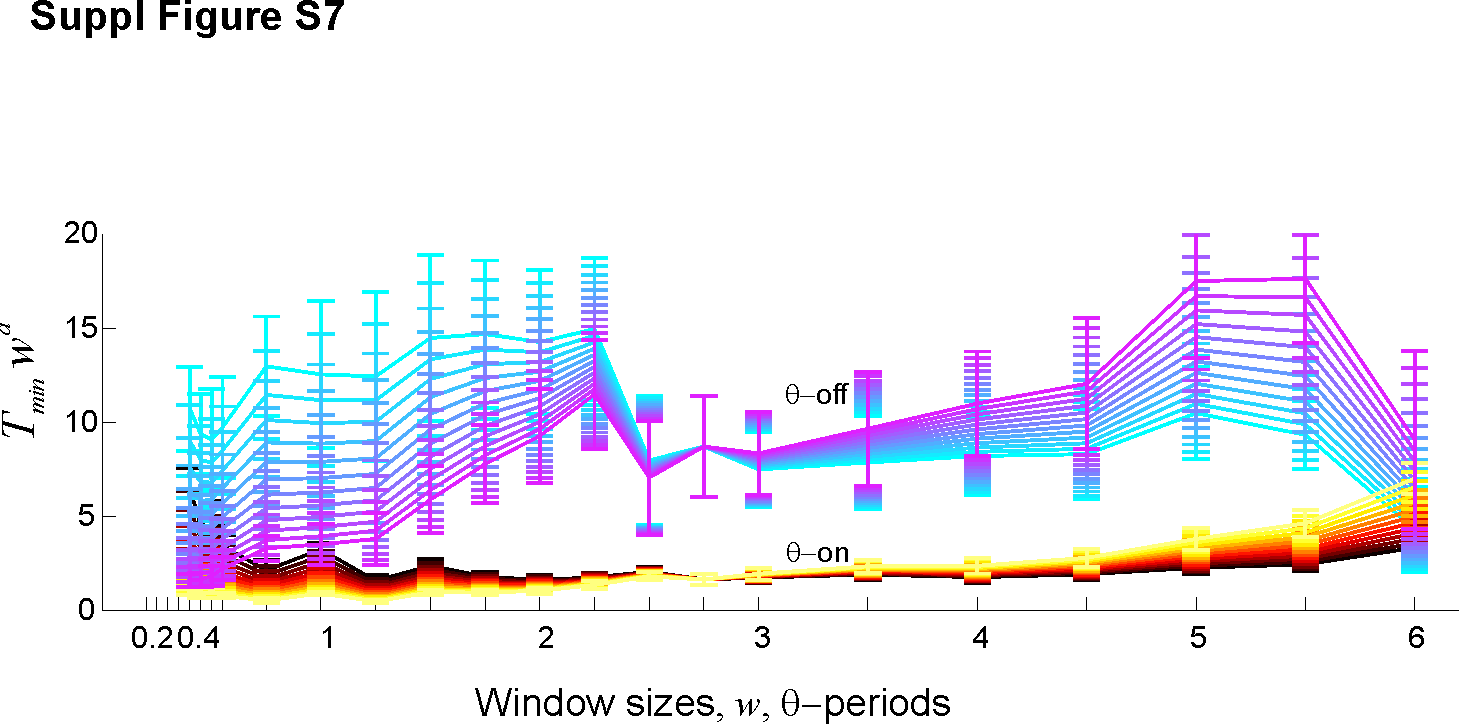

Supplement: Figure S7 — Learning time is inversely proportional to a power of the window width. The test of the hypothesized inverse proportionality dependence between the learning time, Tmin, and the window width, w, Tmin = C/wα, where α and C are constants (see Figure 5 ). To test this hypothesis, we selected the maps that converged for at least 19 out of 24 values of w, and computed the product Tmin wα for 12 values of α taken from the interval 1<α<2. The results show that the product Tmin wα remains bounded for the entire range of α values. While in the θ-off case (colormap “cool”) the variation of the product Tmin wα remains large, in the θ-on case (colormap “hot”) it is nearly constant, which suggests that a nearly hyperbolic relationship Tmin wα = C holds in both cases. The smallest variation is achieved for α = 1.4 in the θ-on case and α = 1.28 in the θ-off case. Notice that as α varies between 1 and 2, the arms of the curve flip so the “most horizontal” position is achieved for 1<α Δ<2. (TIF) [file pcbi.1003651.s007.tif]

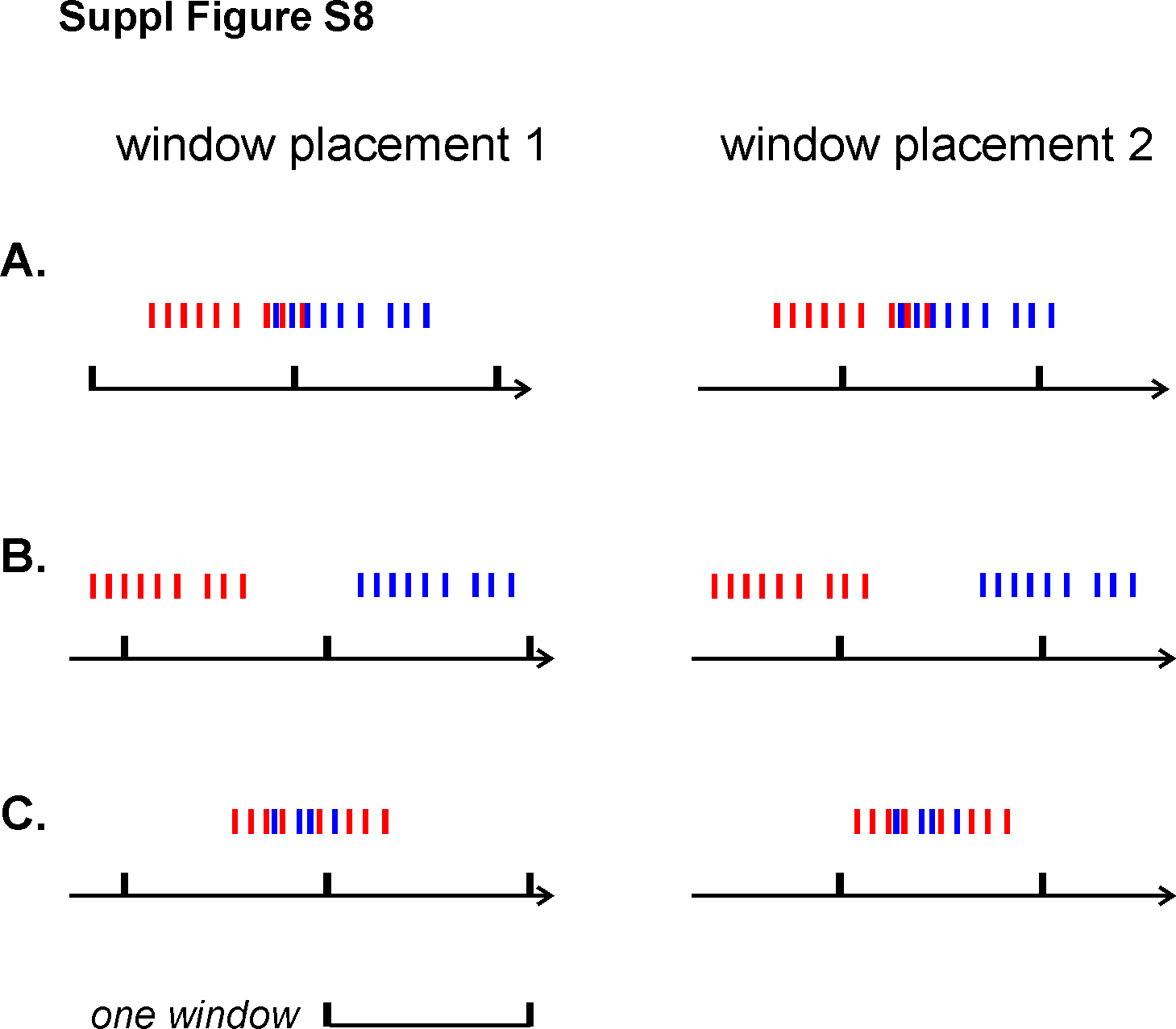

Supplement: Figure S8 — Window widths define what counts as co-activity. While overlap patterns A and C do not depend on the position of the time bins, activity of cells in close temporal succession might, in theory, be interpreted as coactivity or not depending on the position of the time bins and/or the size of the window width (B). In our model, however, we find that window width is the sole determinant of co-firing at the level of neuronal ensemble activity. See Methods. (TIF) [file pcbi.1003651.s008.tif]

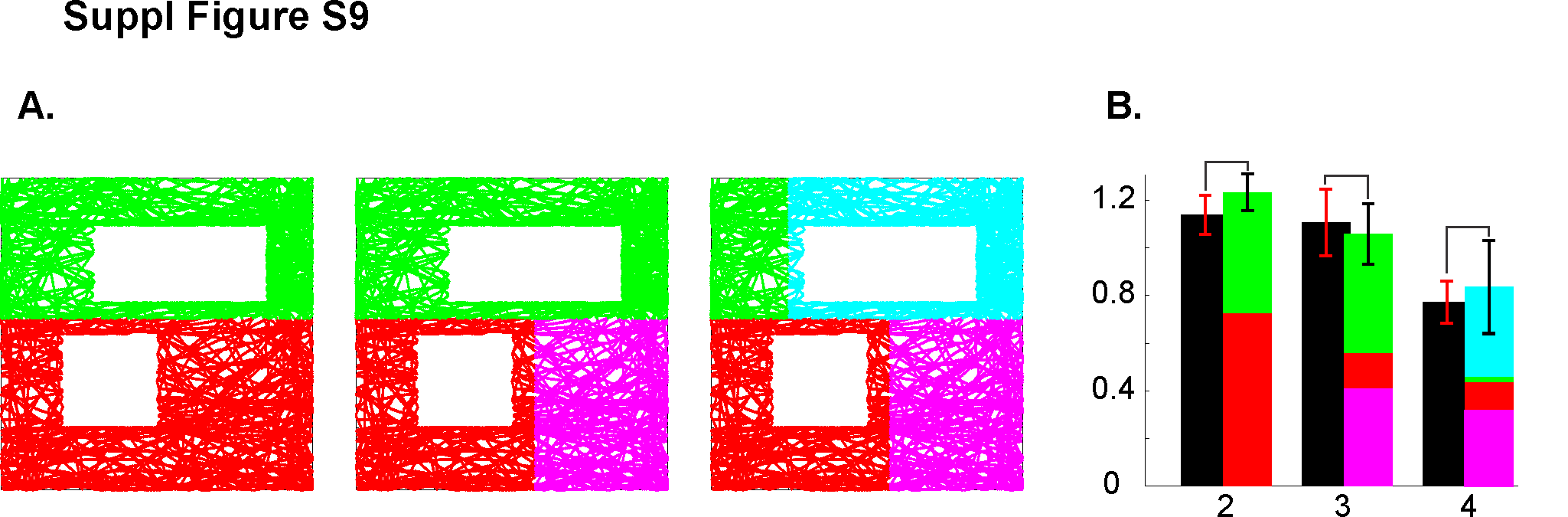

Supplement: Figure S9 — The Mayer-Vietoris Theorem allows us to divide maps into sections to reduce computational cost. A. An area with two holes is divided into two, three or four pieces. We selected the maps that converged to correct topological information in the full arena and in each one of the pieces in which the arena is split, in at least seven out of ten repetitions. B. We then computed the mean learning times Tmin in the full arena (shown by the black bars) and the divided learning times in each piece, Tp,min, represented by colored bars. Colors on the bars correspond to the region colors. There was no statistically significant difference between the bars. (TIF) [file pcbi.1003651.s009.tif]

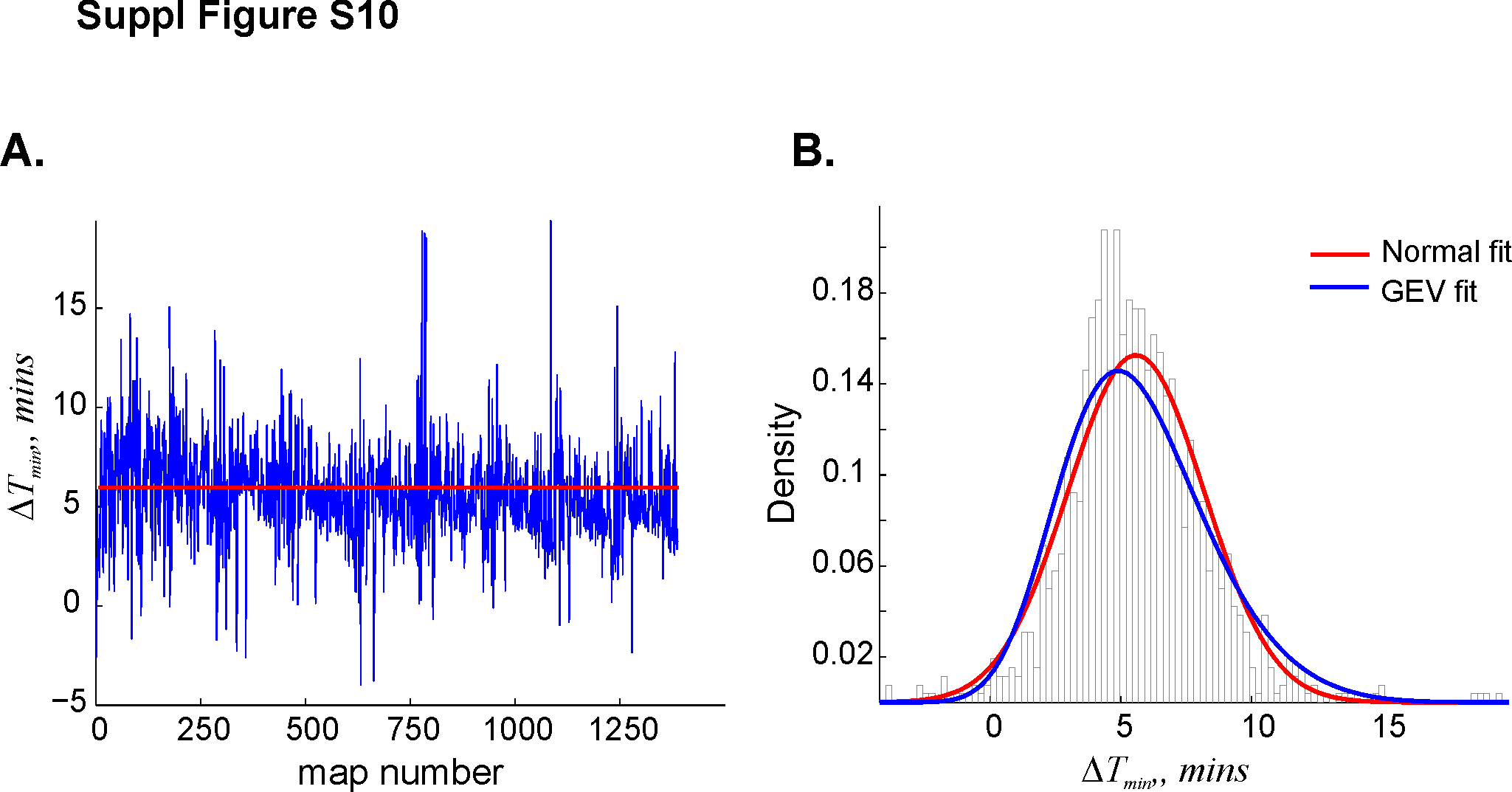

Supplement: Figure S10 — Place field formation time adds to the learning time. A. Differences in learning times in the ensembles with static place fields versus dynamic place fields. Red line marks the mean difference, approximately 5 minutes, which is 120% of the place field formation τ. (Experimentally derived place field formation takes approximately 4 minutes.) B. The distribution of the differences shown in panel A, fit with normal distribution (red) with the mean μ = 5.58 and variance σ = 6.83, and with the GEV distribution (blue) with mean μ = 5.66, variance σ = 7.88. (TIF) [file pcbi.1003651.s010.tif]
